# Novel 1,3-Thiazole Analogues with Potent Activity against Breast Cancer: A Design, Synthesis, In Vitro, and In Silico Study

**DOI:** 10.3390/molecules27154898

**Published:** 2022-07-31

**Authors:** Manar G. Salem, Dina M. Abu El-Maaty, Yassmina I. Mohey El-Deen, Basem H. Elesawy, Ahmad El Askary, Asmaa Saleh, Essa M. Saied, Mohammed El Behery

**Affiliations:** 1 Pharmaceutical Organic Chemistry Department, Faculty of Pharmacy, Suez Canal University, Ismailia 41522, Egypt; drmanargalal@yahoo.com (M.G.S.); yassmina.ibrahim@icloud.com (Y.I.M.E.-D.); 2 Biochemistry Department, Faculty of Pharmacy, Suez Canal University, Ismailia 415222, Egypt; dina_abouelmouti@pharm.suez.edu.eg; 3 Department of Pathology, College of Medicine, Taif University, P.O. Box 11099, Taif 21944, Saudi Arabia; basemelesawy2@gmail.com; 4 Department of Clinical Laboratory Sciences, College of Applied Medical Sciences, Taif University, P.O. Box 11099, Taif 21944, Saudi Arabia; a.elaskary@tu.edu.sa; 5 Department of Pharmaceutical Sciences, College of Pharmacy, Princess Nourah bint Abdulrahman University, P.O. Box 84428, Riyadh 11671, Saudi Arabia; asali@pnu.edu.sa; 6 Chemistry Department, Faculty of Science, Suez Canal University, Ismailia 41522, Egypt; 7 Institute for Chemistry, Humboldt Universität zu Berlin, Brook-Taylor-Str. 2, 12489 Berlin, Germany; 8 The Division of Biochemistry, Chemistry Department, Faculty of Science, Port Said University, Port Said 42526, Egypt

**Keywords:** 1,3-thiazole, 2-hydrazinyl-1,3-thiazole, breast cancer, anticancer activity, antiproliferation, VEGFR-2 kinase activity, apoptosis, cell cycle arrest, molecular docking

## Abstract

Breast cancer is the most common cancer in women, responsible for over half a million deaths in 2020. Almost 75% of FDA-approved drugs are mainly nitrogen- and sulfur-containing heterocyclic compounds, implying the importance of such compounds in drug discovery. Among heterocycles, thiazole-based heterocyclic compounds have demonstrated a broad range of pharmacological activities. In the present study, a novel set of 1,3-thiazole derivatives was designed and synthesized based on the coupling of acetophenone derivatives, and phenacyl bromide was substituted as a key reaction step. The activity of synthesized compounds was screened against the proliferation of two breast cancer cell lines (MCF-7 and MDA-MB-231). Almost all compounds exhibited a considerable antiproliferative activity toward the breast cancer cells as compared to staurosporine, with no significant cytotoxicity toward the epithelial cells. Among the synthesized compounds, compound **4** exhibited the most potent antiproliferative activity, with an IC_50_ of 5.73 and 12.15 µM toward MCF-7 and MDA-MB-231 cells, respectively, compared to staurosporine (IC_50_ = 6.77 and 7.03 µM, respectively). Exploring the mechanistic insights responsible for the antiproliferative activity of compound **4** revealed that compound **4** possesses a significant inhibitory activity toward the vascular endothelial growth factor receptor-2 (VEGFR-2) with (IC_50_ = 0.093 µM) compared to Sorafenib (IC_50_ = 0.059 µM). Further, compound **4** showed the ability to induce programmed cell death by triggering apoptosis and necrosis in MCF-7 cells and to induce cell cycle arrest on MCF-7 cells at the G1 stage while decreasing the cellular population in the G2/M phase. Finally, detailed in silico molecular docking studies affirmed that this class of compounds possesses a considerable binding affinity toward VEGFR2 proteins. Overall, these results indicate that compound **4** could be a promising lead compound for developing potent anti-breast cancer compounds.

## 1. Introduction

With more than 2.3 M diagnosed cases and 685 K deaths in 2020, breast cancer is considered the most globally prevalent cancer (World Health Organization, WHO 2021). Breast cancer treatment is mainly based on surgery followed by radiation therapy to the lymph nodes and breast to reduce the probability of recurrence [1,2,3,4,5]. Additionally, several conservative systematic therapies are based on anticancer drugs, including chemotherapy, antibody (targeted biology) therapy, and hormone (endocrine) therapy. These drugs are mainly used to minimize the possibility of metastasis. Nevertheless, most of the developed alternative systematic therapies are not fully efficient against breast cancer. Further, side effects might include osteoporosis, cardiovascular problems, and induced drug resistance. Accordingly, there is an urgent demand for continuous progress in the discovery and development of novel, safe, and effective lead compounds for long-term cancer therapy with less side effects [6,7,8,9]. 

**Figure 1:**
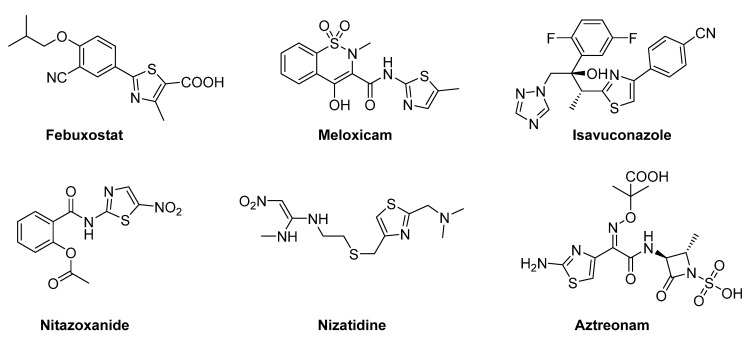
Selected structures of 1,3-thiazole-based FDA-approved drugs.

Heterocyclic compounds have been widely explored in natural products and medicinal chemistry as biologically active scaffolds with significant pharmacological impacts [10,11,12,13,14]. Among heterocyclic compounds, thiazole-based heterocycles have demonstrated a wide range of biological activities and are considered the most common class of heterocycles frequently utilized in drug design and synthetic chemistry. 1,3-Thiazoles are a class of five-membered aromatic heterocyclic rings that contain sulfur and nitrogen as heteroatoms. They are found as constituents of animal cells and in the main scaffold of several natural products including vitamins, alkaloids, and pigments [15]. They are also found as a part of 18 clinically approved drugs (FDA-approved) including antitumor drugs (epothilone, tiazofurin), anti-inflammatory drugs (meloxicam), antifungal drugs (isavuconazole), antiparasitic drugs (thiabendazole, nitazoxanide), antigout drugs (febuxostat), antithrombotic drugs (edoxaban), antiulcer drugs (nizatidine, famotidine), and antibacterial drugs (aztreonam, sulfathiazole, cefepime, and ceftriaxone) (Figure 1) [16,17,18]. The pharmacological significance of the 1,3-thiazole-based compounds has attracted great attention to the design and synthesis of several 1,3-thiazole derivatives with promising pharmacological potential including antiviral, antibacterial, antidiabetic, antioxidant, anti-inflammatory, anticancer, analgesic, antiprotozoal, and antifungal activities [15]. The facile chemical attainability and the possibility of structural optimization make thiazole-based scaffolds the most interesting heterocycles in synthetic medicinal chemistry [19]. 

**Figure 2:**
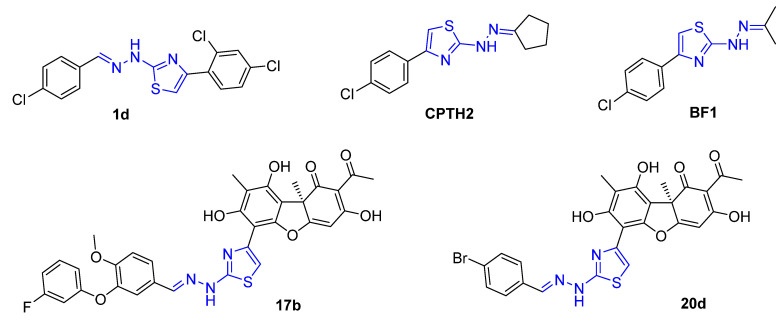
Selected structures of bioactive 2-(2-hydrazinyl)-1,3-thiazole compounds.

Among 1,3-thiazole analogues, 2-(2-hydrazinyl)-1,3-thiazoles demonstrated a broad range of therapeutic applications, including antitumor, antiplasmodial, antiviral, and antimicrobial activities (Figure 2) [20,21,22,23]. Accordingly, 2-(2-hydrazinyl)-1,3-thiazoles chemistry has attracted considerable attention from several researchers. Santana et al. reported the antitumor activity of novel 2-(2-hydrazinyl)-1,3-thiazole derivatives and showed that this class of compounds induces the cell cycle arrest at the G1 stage but also induces mitochondrial depolarization (Figure 2, **1d**) [24]. The continuous efforts by the Lavrik group led to the discovery of hydrazinothiazole derivatives of usnic acid with a nanomolar activity range toward the tyrosyl-DNA phosphodiesterase 1 activity (Figure 2, **20d**, **17b**) [25,26]. Further, Chimenti et al. showed that the anticancer activity of a set of 2-(2-hydrazinyl)-1,3-thiazole analogues attributed to their potent inhibitory activity toward histone acetylase activity (Figure 2, **CPTH2**) [27]. In further studies by the same group, it was demonstrated that this class of compounds exhibits antiproliferative activity in glioblastoma and neuroblastoma cells and inhibits p300 and G_CN5p_ members of histone acetylase protein (Figure 2, **BF1**) [28]. 

**Figure 3:**
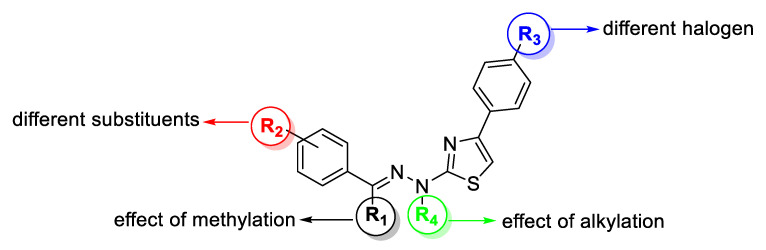
Illustration of the design of 4-substituted-2-hydrazineylthiazole derivatives showing the different structural features examined.

The upregulation of several protein receptors has been reported in breast cancer, including vesicular endothelial growth factor receptor-2 (VEGFR-2), progesterone, and estrogen receptors. Therefore, targeting these receptors has been considered as a potential therapeutic strategy for the development of anti-breast cancer agents [29,30,31,32]. The pharmacological inhibition of VEGFR-2 significantly enhanced apoptosis in breast cancer cells and reduced their proliferation [33,34]. Based on the above-mentioned facts and in continuation of our efforts in synthesizing bioactive molecules [35,36,37,38,39,40,41,42], the presented study aims to design, synthesize, and characterize a novel set of 1,3-thiazole analogues. The design of compounds was envisioned to explore some novel structural features in the 2-hydrazinyl-1,3-thiazole scaffold that could be beneficial for the anti-inflammatory activity of this class of compounds (Figure 3). We screened the anti-proliferative activity of the synthesized compounds to different breast cancer cell lines (MCF-7 and MDA-MB-231), and explored the mechanistic insights responsible for the cytotoxic activity by assessing the cell cycle arrest, programmed cell death, and inhibitory activity toward VEGFR-2 activity. Finally, we performed a detailed in silico molecular modeling study to examine the binding affinity of this class of compounds toward the binding cavity of the VEGFR-2 (PDB code: *2oh4*). 

## 2. Results and Discussion

### 2.1. Chemistry

**Scheme 1:**
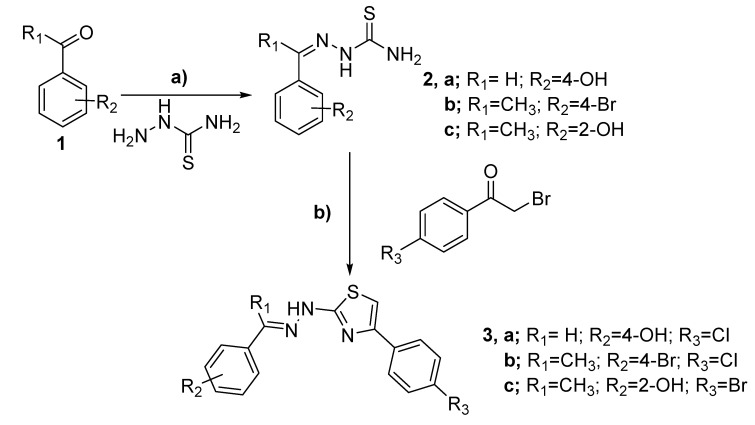
**Synthesis of novel 1,3-thiazole analogues (3a**–**c)**. Reagent and conditions: (**a**) AcOH, EtOH, reflux, 16 h; (**b**) fused AcONa, EtOH, reflux, 12 h.

**Scheme 2:**
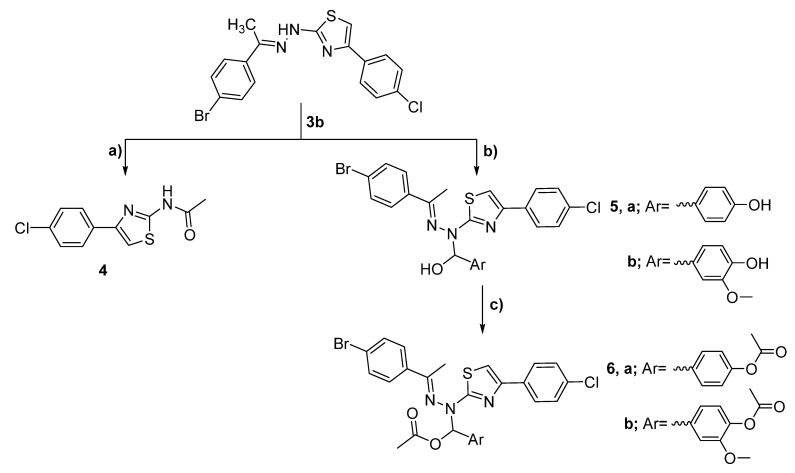
**Synthesis of novel 1,3-thiazole analogues (4**–**6)**. Reagent and conditions: (**a**) Ac_2_O, reflux, 3 h; (**b**) 4-hydroxy benzaldehyde or 3-methoxy-4-hydroxy-benzaldehyde, cat. piperidine, DMF, reflux, 10 h; (**c**) Ac_2_O, reflux, 6 h.

In the present study, a set of 4-substituted-2-hydrazineylthiazole derivatives was synthesized following the synthetic approach outlined in Scheme 1 and Scheme 2. The key step of the 2-hydrazineylthiazole-ring construction relies on the reaction of the thiosemicarbazone to the phenacyl bromide. As illustrated in Scheme 1, the synthesis starts with the condensation of commercially available 4-hydroxybenzaldehyde, 4-bromo-acetophenone, and 2-hydroxy-acetophenone (**1a**–**c**) with thiosemicarbazide in the presence of an acid catalyst under standard conditions to provide the corresponding thiosemicarbazone derivatives (**2a**–**c**) in good yields [40,43,44,45,46]. The obtained thiosemicarbazone derivatives (**2a**–**c**) were subsequently cyclized with 4-substituted phenacyl bromide in the presence of fused sodium acetate under reflux to successfully afford 4-substituted-2-hydrazineyl-1,3-thiazoles (**3a**–**c**) [23,47,48]. 

To further investigate the structural features of the 2-hydrazineyl-1,3-thiazole scaffold, compound **3b** was selected and further derivatives were designed and synthesized. As shown in Scheme 1, acetylation of compound **3b** with acetic anhydride led to the 1-(4-bromophenyl)-ethyl-amine group leaving to afford 2-[5-(4-chlorophenyl)-1,3-thiazole-2-yl]-1-acetyl hydrazine (**4**). Further, the reaction of compound **3b** with commercially available aromatic aldehydes (namely 4-hydroxybenzaldehyde and 3-methoxy-4-hydroxybenzaldehyde) in the presence of catalytic piperidine under reflux yielded the corresponding (*E*)-2-substituted-4-[(2-(1-(4-bromophenyl)-ethylidene]-1-[4-(4-chlorophenyl)-thiazole-2-yl-hydrazinyl](hydroxymethy]-phenols (**5a,b**). In order to explore the role of the 2^nd^ hydroxyl group and/or the phenolic group, compounds **5a,b** were further acetylated with acetic anhydride to afford the corresponding di-acetoxy derivatives (**6a,b**) (Scheme 2). The structure of all synthesized compounds was characterized by different analytical techniques, including melting point, IR, mass spectroscopy, ^1^H-NMR, and ^13^C-NMR analysis. 

#### 2.1.1. Investigation of the NMR Spectra of Synthesized 1,3-Thiazole Derivatives

##### 4-[(2-(4-(4-Chlorophenyl) thiazol-4-yl) hydrazineylidene) methyl]phenol (**3a**)

The ^1^H-NMR spectrum of compound **3a** exhibited douplet signals at δ 6.83–6.85, 7.46–7.48, 7.50–7.52, and 7.86–7.88 ppm due to the eight 8H of the aromatic ring. In addition, the ^1^H-NMR spectrum displayed two singlet signals at δ 7.53 and 7.96, referring to the H-4 of thiazole ring and azo methine (CHN) (Supplementary Figure S1a). The ^13^C-NMR spectrum showed three carbon signals at δ 169.01, 142.64, and 104.50, referring to the C-2, C-4, and C-5 of thiazole ring; respectively. The carbon signals of C-O, C=N, and C-CL were observed in the ^13^C NMR spectrum at δ 159.29, 149.64, and 134.06 ppm. The ^13^C-NMR spectrum showed the carbon signals of the aromatic ring in the region at δ 132.34–116.21 ppm (Supplementary Figure S1b).

##### 2-(2-(1-(4-Bromophenyl)ethylidene)hydrazineyl)-4-(4-chlorophenyl)thiazole (**3b**)

From the current study, the ^1^H-, ^13^C-NMR spectra of compound **3b** showed the structure of this compound in thiazole–thiazolidine tautomers (Supplementary Figure S2d). The ^1^H-NMR spectrum of compound **3b** showed that the presented two characteristic singlet signals at δ 7.42 and 11.38 ppm refer to the H-4 of the thiazole ring and NH-group of compound **3b** for the thiazole form. In addition, the ^1^H-NMR spectrum of compound **3b** exhibited two signals at δ 7.56–7.58 ppm as a douplet signal due to the H-4 of thiazole and singlet signal at δ 10.30 ppm assigned to the proton of the NH-group of the thiazolidine form. Additionally, the ^1^H-NMR spectrum of compound **3b** exhibited four douplet signals at δ 7.47–7.49, 7.62–7.64, 7.72–7.74, and 7.89–7.91 ppm due to the eight protons of two aromatic rings of the two isomers. Methyl protons were observed in the ^1^H-NMR spectrum of compound **3b** at δ 2.29 and 2.32 ppm to support the presence of two isomers (Supplementary Figure S2a). The ^13^C-NMR spectrum of compound **3b** (Supplementary Figure S2b) supported the formation of two isomers of this compound, as it showed the presence of six carbon signals at δ 179. 43, 170.25, 149.93, 147.07, and 14.34, 14.27 ppm due to the C-2 of thiazole, C=N, and CH_3_ groups of two isomers.

##### 2-[1-(2-(4-(4-Bromophenyl)thiazol-2-yl)hydrazineylidene)ethyl]-phenol (**3c**)

The study of the ^1^H-NMR spectrum of compound **3c** showed the presence of two *E* and *Z* stereoisomers of the compound (Supplementary Figure S3d). The ^1^H-NMR spectrum of compound **3c** revealed the presence of two signals at δ 2.43 and 2.49 of the methyl group of two isomers and the hydroxyl (OH), and amion (NH) groups were observed at δ 4.24–4.27 as broad singlet signals, as it formed the hydrogen bond. The aromatic protons of compound **3c** were observed at δ 6.90–6.95 (m.), 7.26–7.30 (t), 7.56–7.59 (d), 7.62–7.64 (d), and 7.83–7.85 (d) due to the eight protons of the aromatic ring. The H-4 of the thiazole ring appeared as a singlet signal at δ 7.43 ppm of two isomers, which was confirmed from the calculation of the integration of hydrogen protons equal to 0.25–0.27 of each proton (Supplementary Figure S3a). Additionally, the ^13^C-NMR spectrum of compound **3c** confirmed the presence of two stereoisomers (*E* and *Z*). The ^13^C-NMR spectrum showed the carbon signals at δ 170.58, 170.24, 166.67, 162.80, 162.50, 159.06, 158.88, 149.27, 148.72, and 147.94, referring to the carbons of thiazole, C-O, and C=N groups. In addition, the ^13^C-NMR spectrum of compound **3c** (Supplementary Figure S3b) exhibited two carbon signals at δ 14.63 and 14.50 ppm, referring to the methyl group of two isomers.

##### 2-[5-(4-Chlorophenyl)-1,3-thiazol-2-yl]-1-acetylhydrazine (**4**)

The ^1^H-NMR spectrum of compound **4** (Supplementary Figure S4a) confirmed the presence of the structure of compound **4** in *E* and *Z* isomers as shown in Supplementary Figure S4d. The ^1^H-NMR spectrum of compound **4** showed two singlet signals at δ 11.14 and 2.16 ppm due to the protons of methyl (CH_3_) and NH groups. In addition, the aromatic protons appeared at δ 7.49–7.51 and 7.92–7.94 ppm as douplet signals, while the H-4 of the thiazole ring was observed at δ 7.83 ppm. From the calculation of integration, the proton integral was found to be equal to 0.49–0.50. The ^13^C-NMR spectrum of compound **4** supported the presence of the *E* and *Z* isomers. The ^13^C-NMR spectrum showed two carbon signals at δ 21.4 and 21.03 ppm, referring to the methyl (CH_3_) group in the two isomers (*E* and *Z* isomers).

##### N-Substituted-1,3-thiazole Derivatives (**6a** and **6b**)

The compounds **6a** and **6b** were obtained from compounds **5a** and **5b** via boiling with acetic anhydride to confirm the structure of compounds **5a** and **5b** (Supplementary Figure S7a). The ^1^H-NMR spectra of compounds **6a** and **6b** showed the structure of these compounds in two stereoisomers (*E* and *Z* isomers) (Supplementary Figure S7d). The ^1^H-NMR spectra of Compounds **6a** and **6b** revealed the presence of two methyl groups in three different isomers, because it gave three singlet signals of each methyl group at δ 2.08–2.22, δ 2.27–2.30, and δ 2.54–2.60 ppm. In the case of compound **6b**, the protons of the methoxy group (OCH_3_) appeared at δ 3.74 and 3.82 of two isomers. In addition, the ^1^H-NMR spectra of compounds **6a** and **6b** showed the different singlet doublet and multiplet signals in the region at δ 6.91–7.95 ppm due to the protons of aromatic rings and H-4 of thiazole. The integral calculation of the number of protons of aromatic and H-4 of thiazole confirmed the structures in *E* and *Z* isomers. In addition, the ^1^H NMR spectra of compounds **6a** and **6b** showed the absence of OH signals of a secondary alcohol, which appeared at δ5.71 in the ^1^H-NMR spectra of compounds **5a** and **5b**. Further, the ^1^H-NMR spectra of compounds **6a** and **6b** exhibited singlet signals at δ 11.07 and 11.05 ppm due to the proton of benzal (Ar-C**H**(N)OCOH_3_), which could be attributed to the deshielding of the benzal proton in compounds **6a** and **6b** more than the proton of benzal in compounds **5a** and **5b**.

#### 2.1.2. Mass Spectrometry Studies

**Scheme 3:**
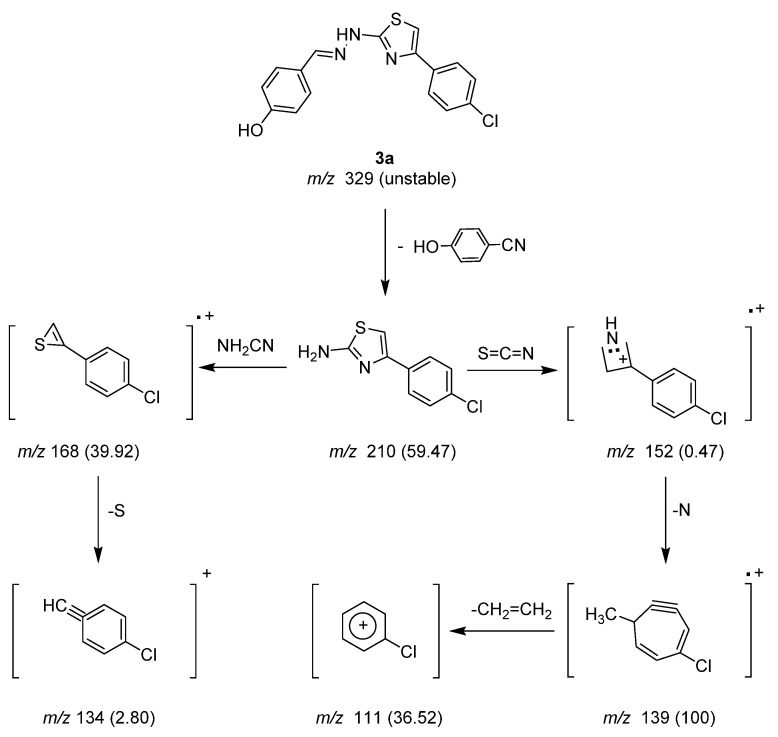
The represented mass fragmentation pattern of compound **3a**.

The mass spectral decomposition modes of the synthesized heterocyclic compounds containing the 1,3-thiazole ring were investigated. The mass spectrum of thiazole derivatives (**3a, 3b** and **3c**) showed intense molecular ion peaks at *m*/*z* 329 (unstable), 405, and 387, corresponding to the molecular formulas C_16_H_12_N_3_ClOS, C_17_H_13_BrN_3_ClS, and C_17_H_14_BrN_3_ClS, respectively. The molecular ion peak of compound **3a** at *m*/*z* 329 (Supplementary Figure S1c) underwent fragmentation to produce a peak at m/z 210 by losing 4-cyanophenol molecules. The loss of (S=C=N) and cyanoamine (NH_2_-CN) from the ion m/z 210 resulted in the ions at *m*/*z* 152 and 168, respectively. The stable ion peak at m/z 139 was observed by the loss of nitrogen atom from the ion at *m*/*z* 152. The stable fragment ion was cleaved to give fragmentation at *m*/*z* 111 (Scheme 3).

**Scheme 4:**
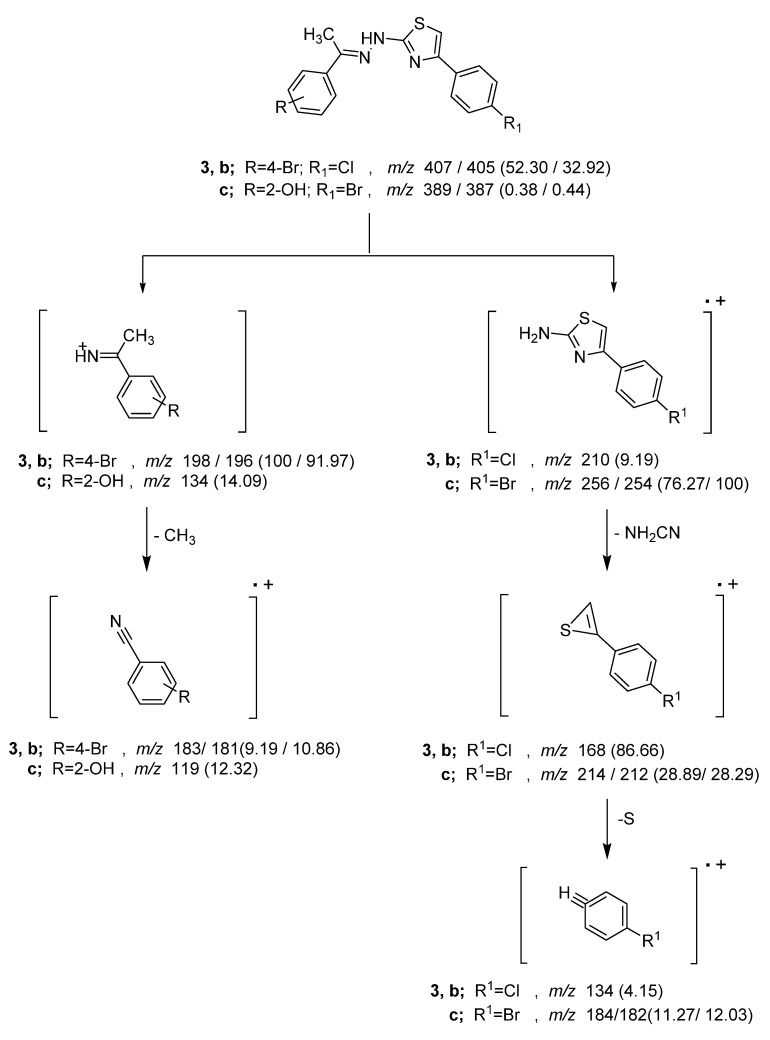
The represented mass fragmentation pattern of compounds **3b** and **3c**.

The molecular ion peak of compounds **3b, c** (Scheme 4) underwent fragmentations to produce a peak ion at *m*/*z* 210 for compound **3b** and a stable ion peak at *m*/*z* 198 for the compound **3c** by losing (4-bromophenylethylidene)amino and (2-hydroxyphenylethylidene) amino fragments, respectively. It further underwent the loss of NH_2_CN and sulfur atoms to give peaks at *m*/*z* 168, 212, and 134, 182, respectively. The molecular ion of compounds **3b** and **4b** (Supplementary Figures S2c and S3c) was also found to undergo fragmentation to produce a stable peak at *m*/*z* 198 for compound **3b** and *m*/*z* 134 for compound **3c**. The loss of methyl groups (CH_3_) from the ion peaks *m*/*z* 198 and *m*/*z* 134 afforded peaks at *m*/*z* 181 and *m*/*z* 119, respectively (Supplementary Figure S7c).

**Scheme 5:**
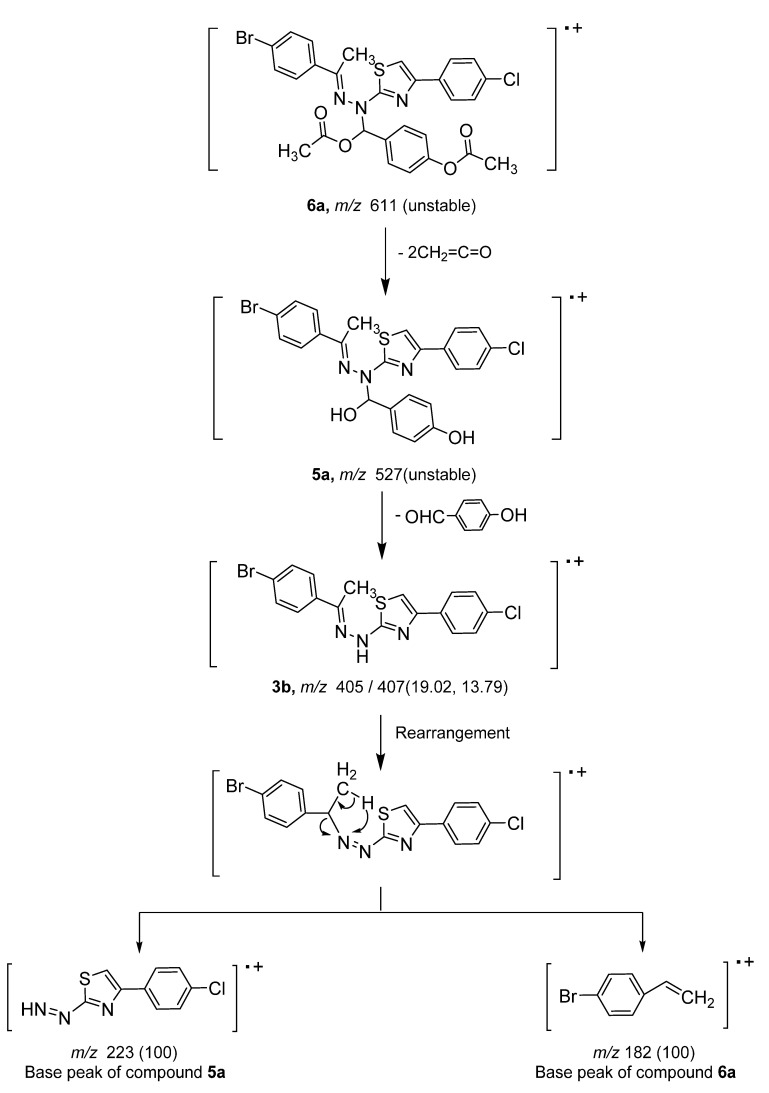
The represented mass fragmentation pattern of compounds **5a** and **6a**.

The molecular ion peak of compound **6a** (unstable), underwent fragmentation to produce a peak at *m*/*z* 407/405, corresponding to the molecular ion peak of compound **3b** by losing two molecules of ketene (2CH_2_=C=O) and 4-hydroxybenzaldehyde, respectively. Meanwhile, in the case of compound **5a**, the mass spectrum showed the molecular ion peak of this compound was unstable. The loss of 4-hydroxybenzaldehyde from the molecular ion peak of compound **5a** (Supplementary Figure S5c) gave a peak at *m*/*z* 405, corresponding to the molecular ion peak for the compound **3b**. The fragmentation of *m*/*z* 407/405 was further fragmented via a pathway similar to compound **3b** (Scheme 5).

**Scheme 6:**
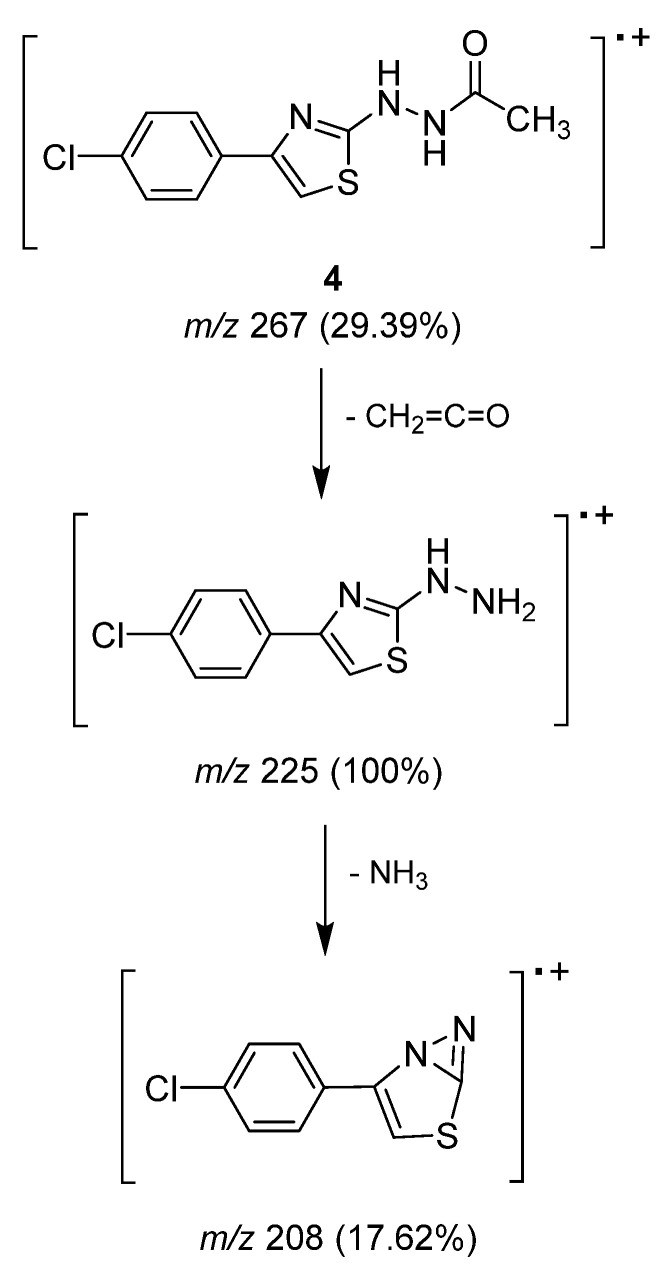
The represented mass fragmentation pattern of compound **4**.

The molecular ion peak at *m*/*z* 267 of 2-[5-(4-chlorophenyl)-1,3-thiazol-2-yl)-1-acetyl hydrazine (**4**) was broken to produce the stable ion peak at m/z 225 (Supplementary Figure S4c) by the loss of the ketene molecule (CH_2_C=O). The loss of the ammonia molecule (NH_3_) from the stable fragment ion *m*/*z* 225 gave the ion peak at *m*/*z* 208. The main fragmentation of compound **4** is illustrated in Scheme 6.

### 2.2. Assessment of Anti-Proliferative Activity against Breast Cancer

**Figure 4:**
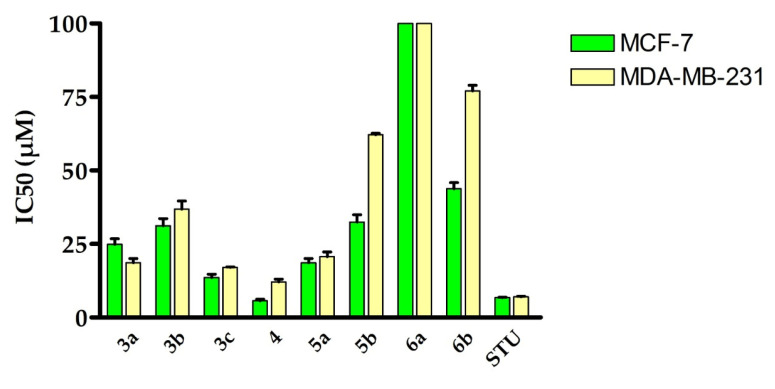
Representative diagram of the anti-proliferative activity of synthesized compounds toward the MCF-7 and MDA-MB-231 breast cancer cell lines.

First, we screened the inhibitory activity of all synthesized compounds (**3a**–**c**, **4**, **5a**–**b**, and **6a**–**b**) against the proliferation of the breast cancer cell lines MCF-7 and MDA-MB-231. Toward this aim, the cells were treated with the synthesized compounds at different concentrations and were incubated for 48 h. In our evaluations, staurosporine (STU) was used as a reference drug. After the cells were washed, they were treated with MTT solution, and an ELISA reader assessed the absorption of the solution at a wavelength of 570 nm. As shown in Table 1, except for compound **6a**, all compounds demonstrated a considerable inhibitory activity toward the proliferation of MCF-7 and/or MDA-MB-231 cells. The 2-benzylidenehydrazineyl)-1,3-thiazole **3a** exhibited a moderate cytotoxic activity (IC_50_ = 24.9 µM and 18.65 µM toward MCF-7 and MDA-MB-231, respectively). The substitution of *p*-phenolic OH with bromide and the introduction of the methyl branch to the 2-benzylidenehydrazineyl significantly reduced the cytotoxic activity (**3b**, IC_50_ = 31.22 µM and 36.84 µM toward MCF-7 and MDA-MB-231, respectively). Interestingly, substituting the chloro- on the *p*-phenyl-thiazole moiety with a bromo- (compound **3c**) and shifting the OH group from the para-position to the ortho-position significantly improved the cytotoxic activity of the compound (IC_50_ = 13.66 µM and 17.1 µM toward MCF-7 and MDA-MB-231, respectively). Further, the replacement of the 2-benzylidenehydrazineyl moiety with the acetyl group (compound **4**) resulted in a significant enhancement in the antiproliferative activity of the compound (IC_50_ = 5.73 µM and 12.15 µM toward MCF-7 and MDA-MB-231, respectively). On the other hand, the condensation of aromatic aldehyde to the 2-(hydrazinyl)-1,3-thiazole moiety resulted in a considerable decrease in the cytotoxicity of the compounds (compounds **5a**, **5b**). Further, acylation of compounds **5a** significantly diminished the activity of the compound as indicated for the cytotoxicity of compounds **6a**, suggesting the role of the second hydroxyl group and/or phenolic group in the cytotoxic activity of compound **5a**. On the other hand, the acylation of compound **5b** has no considerable effect on the cytotoxic activity of the compound (**6b**). We further investigated the cytotoxicity of the compounds toward the epithelial breast cell line MCF-10A. The results revealed that almost all the compounds exhibit no cytotoxic effect, except for compound **4,** which showed a low cytotoxic effect with IC_50_ of 36.83 µM, compared to STU (IC_50_ of 26.72 µM). These results indicate that these compounds possess a considerable and selective cytotoxic activity toward breast cancer cell lines, with a low cytotoxic effect on the epithelial breast cells. Our results are in accordance with previously reported studies, which showed that 1,3-thiazole compounds are potent antiproliferative agents [48,49,50,51,52,53]. Among different investigated compounds, compounds **3c** and **4** demonstrated the most potent anticancer activity toward the examined MCF-7 breast cancer cell line, with an IC_50_ = 13.66 µM and 5.73 µM, respectively, compared to STU, with anIC_50_ of 6.77 µM (Figure 4). Taken together, these findings indicate that the 2-(hydrazinyl)-1,3-thiazole scaffold could be considered for the development of potent anti-breast cancer compounds.

### 2.3. Assessment of VEGFR-2 Kinase Activity

Based on the cytotoxicity results, compounds **3c** and **4** were selected for further investigations. To further explore the mode of action of these compounds, we evaluated the inhibitory activity of compounds **3c** and **4** against VEGFR-2 activity. The vascular endothelial growth factor receptor-2 (VEGFR-2) is a transmembrane tyrosine kinase receptor in mammals that belongs to the family of growth factor receptors and modulates the development of endothelial cells and lymphatic and blood vessels [54,55,56,57]. Several studies have demonstrated the role of the VEGFR-2 receptor in modulating tumor angiogenesis and cancer proliferation. Accordingly, VEGFR-2 has been considered as a potential therapeutic drug target for discovering potent anti-breast cancer agents [56,58,59,60,61,62]. In our study, we were interested in investigating whether the cytotoxic activity of compounds **3c** and **4** is associated with their inhibitory activity toward the VEGFR-2 receptor. To do so, we assessed the inhibitory activity of compounds **3c** and **4,** utilizing sorafenib as a reference drug. As indicated in Table 2, compounds **3c** and **4** showed considerable activity against VEGFR-2 activity. Interestingly, compound **4** (IC_50_ = 0.093 µM) exhibited a significant inhibitory activity toward VEGFR-2 activity compared to sorafenib (IC_50_ = 0.059 µM). These results reveal that the antiproliferative activity of compounds **3c** and **4** could be correlated to their inhibitory activity toward VEGFR-2 activity (Supplementary Figure S15).

### 2.4. Cell Cycle Analysis

**Figure 5:**
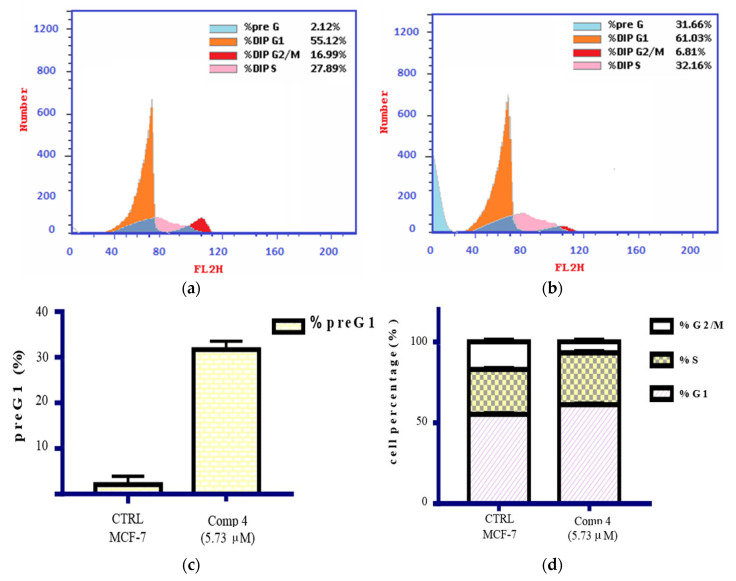
Effect of compound **4** (5.73 µM) on the distribution of MCF-7 cells. (**a**,**b**) Flow cytometry analysis of the different cell cycle phases of treated and untreated MCF-7 cells. (**c**) Effect of compound 4 on the cellular population in the pre-G1 phase. (**d**) Histogram data of the cell cycle distribution of treated and untreated MCF-7 cells.

Based on the anti-proliferative activity results, we selected compound **4** to further explore and achieve insights into the mechanism responsible for the cytotoxicity of this class of compounds. Toward this aim, we assessed the effect of compound **4** on the cell cycle distribution of MCF-7 cells utilizing flow cytometry analysis. The cell cycle distribution analysis was evaluated after incubation of MCF-7 cells with compound **4** at 5.73 µM. As shown in Figure 5, treatment with compound 4 significantly altered the cell cycle distribution of MCF-7 cells. While compound **4** did not significantly affect the cellular population in the S phase, it caused a significant increase in the populations in the pre-G1 phase. Further, compound **4** induced a substantial diminution in the cellular population at the G2/M stage. As indicated in Figure 5a–c, the cellular populations of the G2/M and pre-G1 phases in the control cells were 16.99% and 2.12%, respectively, while the populations were altered to 6.81% and 31.66%, respectively, in the presence of compound 4 at 5.73 µM. These results indicate that the anti-proliferative activity of compound **4** could be attributed to its ability to induce programmed cell death by effectively arresting MCF-7 cells at the pre-G1 phase of the cell cycle while decreasing the cellular population in the G2/M phase. These results are in agreement with previous studies, which showed that 1,3-thiazoles cause cell cycle arrest in HepG2 cells at the S and pre-G1 phases [17,63,64,65].

### 2.5. Apoptosis and Necrosis Analysis

**Figure 6:**
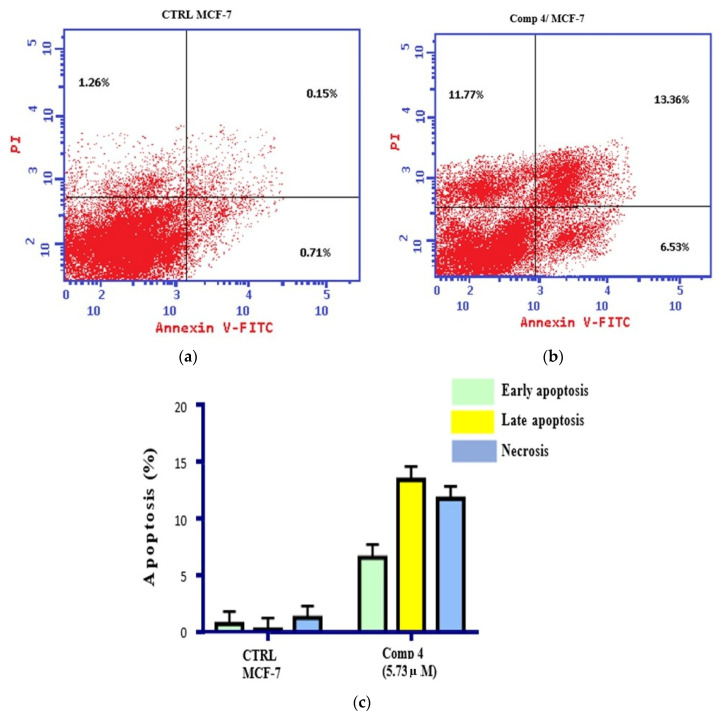
Effect of compound **4** on the programmed cell death of MCF-7 cells. (**a**) Cytofluorometry analysis of untreated MCF-7 cells using Annexin V FITC double labeling assay. (**b**) Cytofluorometry analysis of MCF-7 cells treated with compound **4** at 5.73 µM using Annexin V FITC double labeling assay. (**c**) Graphical representative analysis of the programmed cell death stages on the treated and untreated MCF-7 cells.

Our findings encouraged us to further explore the mechanistic insights accounting for the potent cytotoxicity and cell cycle arrest. To investigate whether the mode of action for the antiproliferative activity of compound **4** toward MCF-7 cells involves apoptosis and/or necrosis, we performed Annexin V- FITC/PI dual staining assay. To this end, we treated MCF-7 cells with compound **4** (5.73 µM) for 24h and assessed the programmed cell death at different stages utilizing the cytofluorometry analysis. The results revealed that compound 4 induced programmed cell death in MCF-7 cells by 32.66% compared to the control cells (2.21%). As demonstrated in Figure 6, compound **4** exhibited a tremendous effect on apoptosis percentage at the early and late stages. Indeed, treatment with compound **4** increased apoptosis by 9 and 89 times at the early and late stages, respectively, compared to control cells. Our findings are in agreement with previous studies, which also reported the potency of 1,3-thiazole analogues to induce the apoptotic pathway in various cancer cell lines [15,17,64]. Interestingly, treatment with compound **4** led to a significant increase in the necrosis percentage (11.77%) by 9 times compared to the control cells (1.26%) (Figure 6). Together, these results indicate that the antiproliferative activity of compound **4** toward MCF-7 cells could be attributed to its ability to induce programmed cell death by triggering both apoptosis and necrosis cell death.

### 2.6. In Silico Molecular Docking Study

**Figure 7:**
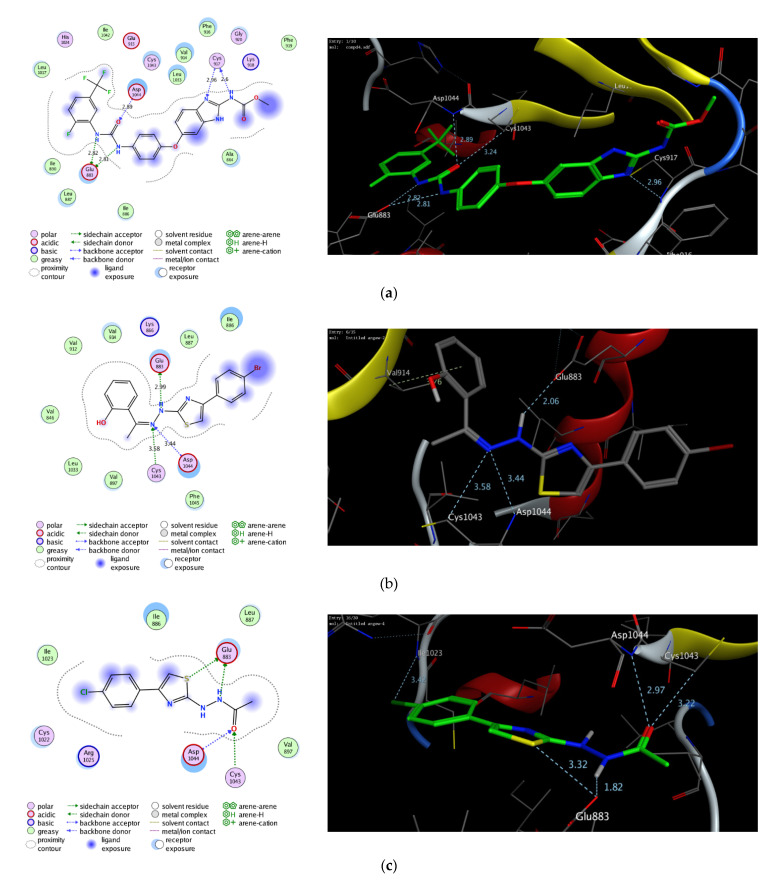
The 2D and 3D molecular docking interactions of the benzimidazole-urea ligand (**a**), compound **3c** (**b**), and compound **4** (**c**) into the binding pocket of VEGFR2 (PDB code: *2oh4*) protein.

To further explore the mechanistic insights into the inhibitory activity of this class of compounds toward VEGFR2 activity, we performed a detailed in silico molecular modeling study aiming at investigating the binding affinity of compounds **3c** and **4** into the binding cavity of the VEGFR2 protein. In the protein database, there are accessible 3D structures of VEGFR2 co-crystallized with different antagonists [66,67,68,69,70,71,72,73,74,75,76,77,78]. The 3D structure (PDB code) was selected based on the ability of the examined compounds to bind to the binding pocket of VEGFR2 in a similar binding pose as the original ligand with a low RMSD value and the resolution of X-ray crystallization. In our investigations, we utilized the X-ray crystal structure of VEGFR2 protein, which has been reported for the design of novel benzimidazole-ureas inhibitors (PDB code: *2oh4*) [71]. The molecular modeling protocol was initially validated by redocking the original benzimidazole-ureas inhibitor before it was utilized to explore the binding affinity of compounds **3c** and **4**. As indicated in Table 3, compounds **3c** and **4** exhibited considerable binding affinity toward the binding pocket of the VEGFR2 receptor (−8.21 and −10.16 kcal/mol, respectively) through a set of interactions. The interactions of compounds **3c** and **4** to the VEGFR2 binding site were thermodynamically favorable as suggested by the docking score values. As shown in Figure 7, this class of compounds binds to the active pocket of the VEGFR2 by forming a set of hydrophilic and hydrophobic interactions with the active amino acid residues together with other amino acid residues in the binding pocket. The original benzimidazole-ureas co-crystallized inhibitor demonstrated a set of hydrophilic interactions with Glu883, Cys917, and Asp104 amino acid residues, together with a set of hydrophobic interactions with greasy amino acid residues in the binding pocket (Figure 7). Compound **3c** exhibited the ability through the hydrazineyl moiety to bind to two of the essential amino acid residues (Asp1044 and Glu883) and additional hydrophilic interaction with the Cys1043 amino acid residues. Further, the binding of compound **3** was further stabilized by the formation of a set of hydrophobic interactions with several greasy amino acid residues. On the other hand, compound **4** revealed the capability to bind to all the essential amino acid residues in the binding site of the VEGFR2 pocket (Glu883, Cys917, and Asp104 residues) through the 2-amino-thiazole moiety and the acetyl group, respectively. The 3D structure of compound **4** displays the ability to bind to Ile1023 residue through the *p*-chloro-substituent. Similarly to compound **3c**, compound **4** could bind to additional amino acid residue (Cys1043) through the acetyl moiety. The binding of compound **4** was also supported by interacting with several greasy amino acid residues (Figure 7). Together, these results reveal that the inhibitory activity of this class of compound toward VEGFR2 activity is associated with their ability to potentially bind to the active pocket of VEGFR2 protein. 

## 3. Materials and Methods

### 3.1. General Description of Materials and Instrumentation

All commercially available chemicals were of analytical grade and were acquired from TCI, Alfa Aesar, and Sigma Aldrich unless otherwise specified. The chemical structure of all synthesized compounds was characterized by elemental analysis, melting point, IR, mass spectroscopy, and ^1^H- and ^13^C-NMR analysis. The melting point of synthesized thiazole derivatives was measured on an electrothermal 200 digital melting point device. The elemental analysis was recorded on a perkin-Elmer 2400 series (Haan, Germany). The IR spectra were recorded as KBr discs utilizing Shimadzu 470 spectrophotometer (Kyoto, Japan). The NMR analysis was performed for a solution in deuterated solvents (DMSO-d6) utilizing a Bruker 400 DRX-Avance spectrometer at 400 MHz and 100 MHz for ^1^H-NMR and ^13^C-NMR, respectively. The chemical shifts are recorded in ppm utilizing tetramethyl silane (TMS) as the internal standard. The mass spectroscopic analysis was performed utilizing Finnigan MATSSQ-7000 mass spectrometer operating at 70 eV.

### 3.2. Synthetic Procedures and Analytic Data of Compounds

#### 3.2.1. General Procedure for the Synthesis of 4-substituted-2-Hydrazinyl-1,3-Thiazole Derivatives (**3a**–**c**)

A solution of thiosemicarbazone derivatives (**2**, 0.01 mol) in 50 mL ethanol was treated with appropriate phenacyl bromide (0.01 mol), followed by fused sodium acetate (0.03 mol). After the resulting reaction mixture was refluxed for 12 h, the mixture was poured into cold water. The precipitate formed was filtered, washed with water, and dried. The crude product was finally recrystallized from an appropriate solvent to afford compound **3**.

##### (*E*)-4-((2-(4-(4-Chlorophenyl)thiazol-2-yl)hydrazineylidene)methyl)-phenol (**3a**)

The entitled compound was obtained as a pale-yellow solid, yield 63%, m.p. 205 °C. FT-IR (KBr)_max_: 3451 (br. OH), 3221 (NH), 1635 (C=N), 1067 (C-O) cm^−1^. ^1^H-NMR (DMSO-*d6*, 400 MHz, ppm): δ 6.84 (d, 2H, *J* = 8.1 Hz, Ar-H), 7.35 (s, 1H, H-4 thiazole), 7.47 (d, 2H, *J* = 8.0 Hz, Ar-H), 7.51 (d, 2H, *J* = 8.1 Hz, Ar-H), 7.87 (d, 2H, *J* = 8.2 Hz, Ar-H), 7.96 (s, 1H, CH=N). ^13^C-NMR (DMSO-*d6*, 100 MHz, ppm): δ 169.00 (C-2 of thiazole), 159.29 (C-O), 149.64 (C=N), 142.46, 134.06, 132,43, 130.56, 129.26, 129.08, 128.47, 127.69, 125.85, 116.21, 104.50 (C-aromatic and thiazole ring). Ms: *m*/*z* (%): 331 (M^+^+2, unstable), 329 (M^+^, unstable), 252 (0.39), 251 (0.81), 250 (4.66), 249 (0.39), 212 (18.89), 211 (11.55), 210 (59.47), 209 (8.40), 170 (13.34), 169 (5.31), 168 (39.92), 167 (6.12), 155 (3.97), 154 (2.71), 142 (1.14), 141 (26.94), 140 (12.00), 139 (100), 138 (16.21), 137 (9.49), 136 (5.17), 134 (2.80), 133 (18.13), 132 (4.83), 125 (5.58), 123 (2.79), 122 (1.76), 121 (3.82), 120 (1.56), 119 (14.19), 113 (11.12), 112 (2.59), 111 (36.52), 102 (5.00), 101 (1.88), 89 (14.82), 87 (2.14), 76 (6.18), 75 (11.61), 74 (3.93), 63 (1.82). Anal. Calcd. for C_16_H_12_N_3_ClOS: C, 58.36; H, 3.65; N, 12.76. Found; C, 58.11; H, 3.33; N, 12.48.

##### (*E*)-2-(2-(1-(4-bromophenyl)ethylidene)hydrazineyl)-4-(4-chlorophenyl)thiazole (**3b**)

The entitled compound was afforded as a yellow solid, yield 67%, m.p. 185 °C. FT-IR (KBr)_max_: 3229 (NH), 1638 (C=N), 1588 (C=C) cm^−1^. ^1^H-NMR (DMSO-*d6*, 400 MHz, ppm): δ 2.29 (s, 3H, CH_3_), 2.32 (s, 3H, CH_3_), 7.41 (s, 1H, H-4 of thiazole), 7.48 (d, 2H, *J* = 8.0 Hz, Ar-H), 7.57 (d, 1H, *J* = 8.0 Hz, H-4 of thiazole), 7.63 (d, 2H, *J*= 8.3 Hz, Ar-H), 7.73 (d, 2H, *J* = 8.1 Hz, Ar-H), 7.91 (d, 2H, *J* = 8.2 Hz, Ar-H) of two isomers, 10.30, 11.38 (br. s, 1H, NH of E/Z isomers). ^13^C-NMR (DMSO-*d6*, 100 MHz, ppm): δ 179.93, 170.25 (C-2 of thiazole), 149.93, 147.07 (C=N), 145.85, 137.52, 137.34*, 134.11, 132.38, 131.84, 131.57, 129.16, 129.12, 128.12, 127.70, 123.18, 122.58, 105.52 (C-aromatic and thiazole of two isomer), 14.34, 14.27* (CH_3_ of two isomers) (* refers to the two isomers). MS *m*/*z* (%): 409 (M^+^+4, 8.99), 408 (M^+^+3, 24.16), 407 (M^+^+2, 52.30), 407 (M^+^+1, 25.66), 405 (M^+^, 32.92), 404 (M^+^-1, 3.70), 274 (2.02), 273 (11.85), 272 (1.50), 271 (6.11), 258 (15.57), 256 (30.96), 254 (2.16), 226 (1.19), 225 (24.25), 224 (9.18), 223 (58.86), 222 (2.68), 214 (2.40), 212 (6.94), 211 (20.00), 210 (4.42), 209 (25.03), 199 (13.28), 198 (100), 197 (25.30), 196 (91.92), 195 (32.35), 184 (18.84), 183 (9.19), 182 (29.96), 181 (10.68), 175 (10.52), 174 (99.54), 173 (9.33), 170 (26.22), 169 (6.23), 168 (86.66), 158 (2.18), 157 (87.71), 156 (9.56), 155 (97.80), 133 (4.15), 132 (15.13), 131 (9.14), 117 (2.50), 116 (5.83), 111 (2.34), 103 (23.05), 102 (44.89), 77 (10.00), 76 (23.41), 75 (16.45), 74 (6.61). Anal. Calcd. for C_17_H_13_N_3_ClBrS: C, 50.31; H, 3.21; N, 10.36. Found: C, 50.18; H, 3.03; N, 10.11.

##### (*E*)-2-(1-(2-(4-(4-Bromophenyl)thiazol-2-yl)hydrazineylidene)ethyl)-phenol (**3c**)

This compound was obtained as an orange solid, yield 71%, m.p. 198 °C. FT-IR (KBr)_max_: 3405 (br. OH), 3221 (NH), 1638 (C=N), 1604, 1582 (C=C), 1121, 1063 (C-O) cm^−1^. ^1^H-NMR (DMSO-*d6*, 400 MHz, ppm): δ 2.43 (s, 3H, CH_3_), 2.49 (s, 3H, CH_3_), 4.24–4.27 (br.s, 1H, OH bonded), 6.90–6.95 (m, 2H, Ar-H), 7.28 (t, 1H, *J* = 7.3 Hz, Ar-H), 7.43 (s, 1H, H-4 of thiazole), 7.57 (d, 1H, *J* = 8.2 Hz, Ar-H), 7.63 (d, 2H, *J* = 8.1 Hz, Ar-H), 7.84 (d, 2H, *J* = 8.2 Hz, Ar-H) of two isomers. ^13^C-NMR (DMSO-*d6*, 100MHz, ppm): δ 170.58, 170.24*, 166.67, 162.80, 162.50*, 159.06, 158.88*, 149.27, 148.72*, 147.94 (C-2 of thiazole, C-O and C=N), 134.24, 132.01, 131.91*, 131.51, 131.17*, 130.04, 129.84, 129.30*, 129.00, 128.02, 127.84, 127.74*, 120.94, 115.71, 115.64*, 105.16 (C-aromatic and Thiazole ring), 14.63, 14.50* (CH_3_ of two isomers) (* refers to the two isomers). Ms: *m*/*z* (%): 389 (M^+^+2, 0.38), 388 (M^+^+1, 0.10), 387 (M^+^, 0.44), 317 (2.02), 316 (2.13), 271 (4.02), 269 (4.44), 258 (5.64), 257 (12.02), 256 (76.27), 255 (58.62), 254 (100), 253 (24.29), 241 (3.07), 239 (3.82), 214 (28.98), 212 (28.29), 211 (3.93), 199 (2.97), 198 (5.07), 197 (2.52), 196 (3.79), 185 (7.05), 184 (11.27), 183 (10.95), 182 (12.03), 175 (5.11), 174 (13.52), 173 (4.23), 157 (5.49), 148 (3.66), 147 (2.03), 146 (2.34), 141 (3.27), 139 (8.56), 138 (6.16), 136 (9.92), 135 (18.14), 134 (14.09), 133 (24.91), 132 (6.30), 121 (15.93), 120 (11.67), 119 (12.32), 106 (1.64), 105 (16.46), 104 (2.98), 103 (4.01), 102 (7.50), 101 (3.40), 92 (7.89), 89 (11.01), 88 (18.43), 82 (14.08), 81 (14.81), 77 (7.13), 76 (6.65), 75 (7.95), 74 (3.72), 65 (2.93), 63 (2.94), 50 (1.89). Anal. Calcd for C_17_H_14_N_3_BrOS: C, 52.71; H, 3.62; N, 10.85. Found; 52.52; H, 3.43; N, 10.61.

#### 3.2.2. Synthesis of N-(4-(4-Chlorophenyl)-Thiazol-2-yl)Acetamide (**4**)

A solution of **3b** (0.01 mol) in acetic anhydride (25 mL) was refluxed for 3h. After the reaction was completed, the mixture was poured into water and kept for 16 h. The obtained solid product was separated by filtration, washed with water, and dried. The crude product was subsequently recrystallized from benzene to provide compound **4**. 

As a pale-yellow solid, yield 61%, m.p. 142–144 °C. FT-IR (KBr)_max_: 3227 (NH), 1695 (C=O), 1631 (C=N), 1605, 1586 (C=C) cm^−1^. ^1^H-NMR (DMSO-*d6*, 400 MHz, ppm): δ 2.16 (s, 3H, CH_3_), 7.50 (d, 2H, *J* = 8.2 Hz, Ar-H), 7.83 (s, 1H, H-4 of thiazole), 7.93 (d, 2H, *J*= 8.0 Hz, Ar-H), 11.14 (s, 1H, NH-CO). ^13^C-NMR (DMSO-*d6*, 100 MHz, ppm): δ 172.21, 170.16, (C=O), 157.90, 147.35, 133.30, 132.89, 129.85, 111.66, 21.03 (C-aromatic and thiazole ring). Ms: *m*/*z* (%): 267 (M^+^, 26.40), 226 (22.66), 225 (100), 224 (23.59), 210 (6.25), 209 (4.17), 208 (17.62), 185 (1.23), 183 (2.03), 174 (3.51), 173 (4.95), 172 (2.58), 168 (2.59), 167 (1.68), 136 (2.87), 134 (4.41), 133 (3.17). Anal. Calcd for C_11_H_9_N_2_ClOS: C, 52.38; H, 3.57; N, 11.11. Found; C, 52.08; H, 3.33; N, 11.06.

#### 3.2.3. General Procedure for the Preparation of 4-[(2-(1-(4- Bromophenyl)-Ethylidene]-1-[4-(4-Chlorophenyl)Thiazole-2-yl)Hydrazinyl](Hydroxy)Methyl]-Phenols (**5a**,**b**)

A solution of compound **3b** (0.01 mol) in dimethyl formamide (30 mL) was treated with aromatic aldehydes (namely; 4-hydroxy benzaldehyde and 3-methoxy-4-hydroxy benzaldehyde, 0.01 mol), followed by a catalytic amount of piperidine (1 mL). The resultant reaction mixture was refluxed for 10 h and subsequently poured into water. The resulting mixture was neutralized with diluted hydrochloric acid (2%) to pH 7 and filtered. The obtained solid was washed with water, dried, and finally re-crystallized with the appropriate solvent to afford compounds **5a** and **5b**.

##### (*E*)-4-((2-(1-(4-Bromophenyl)ethylidene)-1-(4-(4-chlorophenyl)thiazol-2-yl)hydrazineyl)(hydroxy)methyl)phenol (**5a**)

The entitled compound was obtained as an orange solid, yield 71%, m.p. 155–157 °C. FT-IR (KBr)_max_: 3410–3360 (br. OH), 1635 (C=N), 1605, 1591 (C=C), 1121, 1063 (C-O) cm^−1^. ^1^H-NMR (DMSO-*d6*, 400 MHz, ppm): δ 2.29, 2.58 (s, 3H, 2xCH_3_ of two isomers), 5.71 (br. s, 1H, OH of sec. alcohol), 6.79–7.86 (m, 14H, Ar-H, CHO and phenolic OH). ^13^C-NMR (DMSO-*d6*, 100 MHz, ppm): δ 167.93, 157.00, 146.57, 137.43, 136.98, 133.89, 133.38, 132.22, 132.08*, 131.99, 131.86*, 131.65, 130.67, 130.02*, 129.44, 129.21*, 128.73, 128.13, 125.94, 122.61, 116.85, 116.10*, 15.62, 14.43* (CH_3_ of two isomer) (* refers to the two isomers). Ms *m*/*z* (%): 527 (M^+^, unstable), 405 (91.02), 404 (16.30), 393 (2.32), 392 (7.62), 391 (4.20), 390 (6.15), 379 (5.42), 378 (1.19), 377 (2.23), 316 (5.69), 315 (3.01), 314 (1.63), 301 (6.56), 300 (3.08), 258 (2.52), 256 (2.22), 250 (3.75), 225 (17.36), 224 (11.58), 223 (28.71), 222 (24.55) 212 (36.18), 196 (55.93), 195 (15.24), 185 (10.57), 184 (15.94), 183 (19.92), 182 (20.46), 181 (6.90), 175 (5.63), 174 (35.59), 173 (19.89), 170 (26.43), 169 (11.09), 168 (73.01), 167 (8.59), 158 (2.70), 157 (35.21), 155 (39.33), 138 (9.50), 137 (5.30), 133 (13.77), 103 (8.73), 102 (14.16), 89 (7.87), 77 (3.68), 76 (7.34), 75 (9.35). Anal. Calcd for C_24_H_19_N_3_BrClO_2_S: C, 54.65; H, 3.61; N, 7.97. Found; C, 54.45; H, 3.37; N, 7.77.

##### (*E*)-4-((2-(1-(4-Bromophenyl)ethylidene)-1-(4-(4-chlorophenyl)thiazol-2-yl)hydrazineyl)(hydroxy)methyl)-2-methoxyphenol (**5b**)

The entitled compound was afforded as an orange solid, yield 73%, m.p. 158–160 °C. FT-IR (KBr)_max_: 3420–3380 (br. OH), 1638 (C=N), 1605, 1583 (C=C), 1205, 1093 (C-O) cm^−1^. ^1^H-NMR (DMSO-*d6*, 400MHz, ppm): δ 2.29 (s, 3H, CH_3_), 2.59 (s, 3H, CH_3_), 3.71 (s, 3H, OCH_3_), 3.88 (s, 3H, OCH_3_), 5.71 (br. s, 1H, OH of sec.alcohol), 6.81–7.95 (m, 13H, Ar-H, CHO and phenolic OH), 10.11, 11.36 (s, 1H, OH). ^13^C-NMR (DMSO-*d6*, 100 MHz, ppm): δ 178.81, 174.98, 167.82, 167.73*, 150.29, 148.40, 148.29*, 146.40, 137.46, 136.98*, 136.26, 134.66, 133.91, 132.83, 132.69*, 132.22, 132.07, 131.99, 131.86, 130.67, 130.03, 129.72, 129.45, 129.39*, 128.72, 128.12, 126.52, 124.76, 122.59, 120.43, 116.70, 116.10, 115.81, 112.57, 56.26, 56.16* (2xOCH_3_), 15.54, 14.45* (2 × CH_3_ of two isomers) (* refers to the two isomers). Ms: *m*/*z* (%): 557 (M^+^, unstable), 288 (1.17), 287 (1.96), 200 (21.89), 199 (4.03), 198 (23.72), 185 (72.47), 184 (34.93), 183 (100), 182 (22.89), 158 (3.45), 157 (65.24), 156 (22.21), 155 (80.49), 154 (10.61), 140 (1.75), 13 (34.00), 138 (7.36), 137 (26.53), 112 (3.12), 111 (11.34), 104 (41.18), 102 (2.34), 101 (5.66), 87 (3.47), 86 (41.45), 85 (3.62), 77 (3.47), 76 ((25.30), 75 (39.06), 74 (25.66), 73 (7.83), 64 (7.57), 63 (6.43), 51 (12.98), 50 (25.91). Anal. Calcd for C_25_H_21_N_3_BrClO_3_S: C, 53.86; H, 3.77; N, 7.54. Found; C, 53.53; H, 3.48; N, 7.34.

#### 3.2.4. Synthesis of Compounds **6a**,**b**

Compound **5a** or **5b** (0.01 mol) was treated with acetic anhydride 25 mL, and the resulting reaction mixture was refluxed for 6 h. After the reaction was completed, the mixture was poured into ice water and kept for 24 h, during which a solid product started to form. The resultant solid product was separated by filtration, washed with water, and dried. The crude product was finally recrystallized from ethanol to provide compounds **6a** and **6b**.

##### (*E*)-4-(Acetoxy(2-(1-(4-bromophenyl)ethylidene)-1-(4-(4-chlorophenyl)thiazol-2-yl)hydrazineyl)methyl)phenyl acetate (**6a**)

The entitled compound was obtained as a pale orange solid, yield 63%, m.p. 126–128 °C. FT-IR (KBr)_max_: 1752 (C=O), 1631 (C=N), 1605, 1589 (C=C), 1125, 1089, 1063 (C-O) cm^−1^. ^1^H-NMR (DMSO-*d6*, 400 MHz, ppm): δ 2.08 (s, 3H, CH_3_), 2.22 (br-s, 3H, CH_3_), 2.31 (s, 3H, CH_3_), 2.34 (s, 3H, CH_3_), 2.58 (s, 3H, CH_3_), 7.21–7.34 (m, 3H, Ar-H), 7.42 (s, 1H, H-4 of Thiazole), 7.69–7.76 (m, 3H, Ar-H), 7.80 (d, 2H, *J*= 8.1 Hz, Ar-H), 7.89 (d, 2H, *J* = 8.3 Hz, Ar-H), 7.94 (d, 2H, *J* = 8.2 Hz, Ar-H), 11.07 (s, 1H, N-CH(Ar)OCO). ^13^C-NMR (DMSO-*d6*, 100 MHz, ppm): δ 179.01, 174.06, 170.04, 169.43*, 164.76 (C=O and C-2 of thiazole), 152.19 (C-O), 136.91, 136.79*, 136.27, 135.59*, 133.36, 133.27, 133.04, 132.91, 132.66, 132.22, 132.10, 132.05*, 131.36, 130.68, 130.02, 129.53, 129.50, 129.26, 128.96, 124.97, 123.21, 122.90 (C-aromatic and thiazole ring), 21.38, 20.96 (2xOCOCH_3_), 15.72 (CH_3_) (* refers to the two isomers). Ms *m*/*z* (%): 611 (M^+^, unstable), 408 (4.27), 407 (11.87), 406 (8.27), 405 (13.79), 404 (3.51), 360 (1.80), 358 (1.29), 316 (1.34), 315 (2.58), 302 (4.05), 301 (2.57), 300 (3.04), 269 (13.44), 268 (6.60), 267 (42.27), 266 (14.55), 256 (19.99), 255 (4.54), 254 (20.25), 252 (10.23), 242 (2.98), 241 (12.94) 240 (5.03), 239 (20.21), 238 (6.51), 237 (8.41), 227 (26.85), 226 (24.61), 225 (94.00), 224 (40.89), 223 (45.74), 214 (23.83), 213 (17.71), 212 (47.14), 211 (33.14), 210 (54.44), 209 (32.67), 208 (21.13), 199 (49.21), 198 (67.92), 197 (61.77), 196 (75.07), 195 (16.87), 185 (12.08), 184 (55.42), 183 (68.15), 182 (100), 181 (54.33), 180 (31.92), 175 (11.92), 174 (42.70), 173 (22.02), 170 (19.19), 169 (13.88), 168 (43.82), 165 (10.92), 164 (4.88), 163 (13.11), 157 (50.71), 156 (8.95), 155 (51.04), 154 (7.85), 139 (38.71), 138 (28.10), 137 (29.12), 136 (17.94), 134 (19.71), 133 (23.20), 132 (12.75), 131 (9.86), 121 (16.41), 120 (10.06), 119 (12.03), 118 (7.51), 117 (10.97), 116 (18.68), 113 (6.29), 112 (8.06), 111 (15.84), 103 (22.69), 102 (63.39), 101 (20.96), 90 (5.73), 89 (12.48), 77 (10.17), 76 (23.16), 75 (27.83), 74 (8.39). Anal. Calcd. for C_28_H_23_N_3_BrClO_4_S: C, 54.99; H, 3.76; N, 6.87. Found; C, 54.66; H, 3.49; N, 6.66.

##### *(E*)-4-(Acetoxy(2-(1-(4-bromophenyl)ethylidene)-1-(4-(4-chlorophenyl)thiazol-2-yl)hydrazineyl)methyl)-2-methoxyphenyl acetate (**6b**)

The entitled compound was afforded as a pale orange solid, yield 63%, m.p. 126–128 °C. FT-IR (KBr)_max_: 1752 (C=O), 1633 (C=N), 1610, 1591 (C=C), 1130, 1086, 1056 (C-O) cm^−1^. ^1^H-NMR (DMSO-*d6*, 400 MHz, ppm): δ 2.08 (s, 3H, CH_3_), 2.11 (s, 3H, CH_3_), 2.27 (s, 3H, CH_3_), 2.30 (s, 3H, CH_3_), 2.54 (s, 3H, CH_3_), 2.60 (s, 3H, CH_3_), 3.74 (s, 3H, OCH_3_), 3.82 (s, 3H, OCH_3_), 6.92 (d, 1H, *J* = 8.1 Hz, Ar-H), 7.16 (d, 2H, *J*= 8.3 Hz, Ar-H), 7.30–7.49 (m, 3H, Ar-H and H-4 of Thiazole), 7.73 (d, 2H, *J* = 8.2 Hz, Ar-H), 7.89 (d, 2H, *J* = 8.1 Hz, Ar-H), 7.95 (d, 2H, *J* = 8.0 Hz, Ar-H), 11.05 (s, 1H, N-CH(Ar)OCO). ^13^C-NMR (DMSO-*d6*, 100 MHz, ppm): δ 179.04, 174.06, 168.84, 164.56 (C=O and C-2 of thiazole), 151.61 (C-O), 139.17, 136.84, 134.11, 133.29, 132.23, 132.11, 131.43, 130.86, 130.01, 129.53, 128.93, 124.99, 124.17, 123.79, 122.13, 116.66 (C-aromatic and thiazole ring), 56.47 (OCH_3_), 27.11 (CH_3_), 21.29, 20.96 (OCOCH_3_), 15.68 (CH_3_). Ms *m*/*z* (%): 641 (M^+^, unstable), 96 (10.12), 95 (1.94), 91 (1.48), 82 (1.46), 81 (100), 80 (3.74), 71 (1.07), 53 (10.44), 51 (10.44). Anal. Calcd for C_29_H_25_N_3_BrClO_4_S: C, 54.29; H, 3.90; N, 6.55. Found; C, 54.03; H, 3.63; N, 6.22.

### 3.3. Assessment of Anti-Proliferative Activity against Breast Cancer

To assess the anti-proliferative activity of the synthesized compounds toward the different cancer cell lines (MCF-7 and MDA-MB-231) and the epithelial breast cell line MCF-10A, the MTT assay was employed following the previously reported protocol [79,80]. Briefly, cells (10^5^ cells/well) were treated in triplicate at 37 °C with compounds **3a-c**, **4**, **5a-b**, and **6a-b** at different concentrations (DMSO stock solutions) for 48h and in a 5% CO_2_ atmosphere. In our screening, Staurosporin (STU) has been utilized as a reference positive anticancer drug. After the cells were washed, they were subsequently treated with MTT solution (40 µL, MTT 5 mg/mL in 0.9% NaCl) and incubated at 37 °C for 4 h. Finally, the resulting MTT crystals were dissolved at ambient temperature in acidified isopropanol (180 µL/well) with shaking, and the absorption was assessed at 570 nm utilizing ELISA. The obtained data were analyzed, and the inhibitory activity of compounds (IC_50_) toward cell viability of different cell lines was determined and expressed as means ± S.E.M.

### 3.4. Assessment of VEGFR-2 Enzyme Activity

The inhibitory activity of compounds **3c**, and **4** was assessed toward VEGFR-2 activity as previously reported utilizing the human VEGFR-2/KDR ELISA kit (RBMS#2019R) following the manufacturer’s instructions [81]. In our experiments, we used Sorafenib as a positive control drug reference. The activity of compounds was assessed at different concentrations, and the IC_50_ values were determined.

### 3.5. Cell Cycle Analysis

To assess the effect of compound **4** on the cell cycle of MCF-7 cells, we treated the MCF-7 cells with compound **4** and analyzed the cell cycle arrest at different stages as previously reported [82]. Briefly, after MCF-7 cells (3 × 10^5^ cells/well) were incubated for 12 h at 37 °C, the cells were treated with compound 4 (5.73 µM) and incubated for another 24 h at the same temperature. Subsequently, the cells were fixed overnight at 20 °C with 75% ethanol, followed by washing with PBS buffer and centrifugation. After the cells were incubated with propidium iodide (5 mg/mL, Sigma, Ronkonkoma, NY, USA) and Rnase (10 mg/mL, Sigma, Ronkonkoma, NY, USA), cytofluorometry analysis was performed using FACS Calibur cytometer, and the results were analyzed utilizing Cell quest software (BD Bioscience, San Jose, CA, USA).

### 3.6. Annexin V-FITC/PI Dual Staining Analysis

To explore the effect of compound **4** on the programmed cell death mechanisms of MCF-7 cells, fluorescent Annexin V-FITC/ PI assay was performed using flow cytometry following a previously reported procedure [83]. Briefly, MCF-7 cells (2 × 10^5^) were treated with compound **4** at 5.73 µM concentration. After incubation for 24 h, the harvested cells were stained in the dark at 37 °C with Annexin V-FITC/ PI dye for 15 min. Cytofluorometry analysis was assessed by BD *FACS Calibur* (Becton and Dickinson, Heidelberg, Germany), and the BD cell Quest pro software was utilized to analyze the results.

### 3.7. In Silico Molecular Modeling Study

Molecular modeling studies provide a valuable view of ligand–receptor binding and propose the mode of action of bioactive compounds. The biological activities of chemical compounds are attributed to the binding with key amino acids of the active site of cellular target proteins [35,84,85,86,87,88,89,90]. To elucidate the mechanism by which the synthesized compounds conjugate with VEGFR2 activity, a molecular docking tool was used to examine the interactions of the compounds **3c** and **4** toward VEGFR2. The 3D structure of VEGFR2 was obtained from the RCSB Protein Data Bank (http://www.rcsb.org/, accessed on 1 May 2022, VEGFR2 PDB: *2oh4*) [71]. The Chem. Draw tool was used to construct the structure of the target compounds **3c** and **4**. Then, the protonated 3D structure of target ligands **3c** and **4**, the preparation of VEGFR2, and the docking studies were performed using MOE software as previously reported [35,84,85,86,87,88,89,90,91]. The validity of the applied protocol was evaluated by performing a molecular docking of the original inhibitor to affirm the main interactions that exist in the reported crystal structure. 

## 4. Conclusions

We have designed, synthesized, and characterized a set of novel 1,3-thiazole analogues based on the condensation of thiosemicarbazone to substitute phenacyl bromide as a key reaction step. The cytotoxic activity of the synthesized compound was screened for two different cancer cell lines (MCF-7 and MDA-MB.231). Our findings revealed that the synthesized compounds exhibit a considerable antiproliferative activity toward the investigated cancer cell lines without a substantial effect on the proliferation of epithelial cells. Among the investigated compounds, compounds **3c** and **4** demonstrated a significant antiproliferative activity compared to the reference drug. These compounds also showed a substantial inhibitory activity toward VEGFR-2 activity. Our findings revealed that compound **4** exhibits the most potent cytotoxicity and inhibitory activity toward VEGFR-2 activity. Further investigations on compound **4** revealed that compound **4** causes a cell cycle arrest on MCF-7 cells at the G1 stage while decreasing the cellular population in the G2/M phase. Further, compound **4** displayed a tremendous effect on apoptosis percentage at the early and late stages and significantly increased the necrosis percentage, indicating that the antiproliferative activity of compound **4** could be correlated to its ability to induce programmed cell death by triggering both apoptosis and necrosis cell death. Finally, an in silico molecular modeling study affirmed that this class of compounds has a considerable binding affinity toward VEGFR2 protein. Taken together, our study reveals that compound **4** could be a suitable lead compound for the development of potent anti-breast cancer compounds. Further studies should be performed to deeply investigate the mode of action of compound **4** and its applicability in an animal model.

## Figures and Tables

**Table 1:** Inhibitory activity of compounds (**3a**–**c**, **4, 5a**–**b**, and **6a**–**b**) toward the proliferation of various breast cancer cell lines and the epithelial breast cell line.

Compound	IC_50_ (µM)		
	MCF-7	MDA-MB-231	MCF-10A
**3a**	24.9 ± 1.9	18.65 ± 1.42	NT
**3b**	31.22 ± 2.38	36.84 ± 2.81	NT
**3c**	13.66 ± 1.04	17.08 ± 0.13	NT
**4**	5.73 ± 0.44	12.15 ± 0.93	36.83 ± 05
**5a**	18.64 ± 1.42	20.72 ± 1.58	NT
**5b**	32.48 ± 2.47	62.12 ± 0.47	NT
**6a**	>100	>100	NT
**6b**	43.81 ± 2.04	77.04 ± 1.95	NT
**STU**	6.77 ± 0.08	7.03 ± 0.19	26.72 ± 1.26

**Table 2:** Assessment of VEGFR-2 inhibitory activity of 1,3-thiazole derivatives **3c** and **4** compared with sorafenib.

Comp No.	IC_50_ Values (µM)
**3c**	0.253 ± 0.54
**4**	0.093 ± 0.22
**Sorafenib**	0.059 ± 0.35

**Table 3:** Docking scores and interactions of compounds **3c** and **4** with VEGFR2 (PDB: *2oh4*) protein.

Protein (PDB Code)	Compound	Hydrophilic Interactions	Distance (A)	Hydrophobic Interactions	S (kcal/mol)
VEGFR2 (*2oh4*)	benzimidazole-urea ligand	Glu883 Glu883 Cys917 Cys917 Asp1044	2.82 2.81 2.6 2.96 2.89	Ala864, Ile886, Leu887, Ile890, Leu1017, Phe919, Phe916, Val914, Leu1033, Ile1042	−11.47
	**3c**	Glu883 Cys1043 Asp1044	2.99 3.58 3.44	Val846, Ile886, Leu887, Val897, Val912, Val914, Leu1033, Phe1045	−8.21
	**4**	Glu883 Glu883 Ile1023 Cys1043 Asp1044	1.82 3.32 3.42 3.22 2.97	Val897, Leu887, Ile886, Ile1045	−10.16

## Data Availability

Not applicable.

## Supplementary Materials

The following supporting information can be downloaded at: https://www.mdpi.com/article/10.3390/molecules27154898/s1, Figure S1: 1H-NMR spectrum of compound **3a**; Figure S2: Mass spectrum of compound **3a**; Figure S3: NMR spectrum of compound **3b**; Figure S4: Mass spectrum of compound **3b**; Figure S5: NMR spectrum of compound **3c**; Figure S6: Mass spectrum of compound **3c**; Figure S7: NMR spectrum of compound **4**; Figure S8: Mass spectrum of compound **4**; Figure S9: NMR spectrum of compound **5a**; Figure S10: Mass spectrum of compound **5a**; Figure S11: NMR spectrum of compound **5b**; Figure S12: NMR spectrum of compound **6a**; Figure S13: Mass spectrum of compound **6a**; Figure S14: NMR spectrum of compound **6b**; Figure S15: Inhibitory activity of compounds **3c**, **4** and Sorafenib toward the VEGFR-2 kinase activity.

## Sample Availability

Samples of the compounds **3a**–**c 4**, **5a**,**b**, and **6a**,**b** are available from the authors.

## References

[1] Waks A.G., Winer E.P. (2019). Breast Cancer Treatment: A Review. JAMA.

[2] Loibl S., Poortmans P., Morrow M., Denkert C., Curigliano G. (2021). Breast Cancer. Lancet.

[3] Harbeck N., Penault-Llorca F., Cortes J., Gnant M., Houssami N., Poortmans P., Ruddy K., Tsang J., Cardoso F. (2019). Breast Cancer. Nat. Rev. Dis. Primer.

[4] Tong C.W.S., Wu M., Cho W.C.S., To K.K.W. (2018). Recent Advances in the Treatment of Breast Cancer. Front. Oncol..

[5] McAndrew N.P., Finn R.S. (2022). Clinical Review on the Management of Hormone Receptor–Positive Metastatic Breast Cancer. JCO Oncol. Pract..

[6] Arora S., Narayan P., Osgood C.L., Wedam S., Prowell T.M., Gao J.J., Shah M., Krol D., Wahby S., Royce M. (2022). U.S. FDA Drug Approvals for Breast Cancer: A Decade in Review. Clin. Cancer Res. Off. J. Am. Assoc. Cancer Res..

[7] An J., Peng C., Xie X., Peng F. (2022). New Advances in Targeted Therapy of HER2-Negative Breast Cancer. Front. Oncol..

[8] Cui W., Aouidate A., Wang S., Yu Q., Li Y., Yuan S. (2020). Discovering Anti-Cancer Drugs via Computational Methods. Front. Pharmacol..

[9] Quijia C.R., Chorilli M. (2022). Piperine for Treating Breast Cancer: A Review of Molecular Mechanisms, Combination with Anticancer Drugs, and Nanosystems. Phytother. Res. PTR.

[10] Sabir S., Alhazza M.I., Ibrahim A.A. (2016). A Review on Heterocyclic Moieties and Their Applications. Catal. Sustain. Energy.

[11] Ramsden C.A., Ramsden C.A. (2022). Chapter One—Heterocyclic Mesomeric Betaines: An Overview. Advances in Heterocyclic Chemistry.

[12] Kalaria P.N., Karad S.C., Raval D.K. (2018). A Review on Diverse Heterocyclic Compounds as the Privileged Scaffolds in Antimalarial Drug Discovery. Eur. J. Med. Chem..

[13] Kerru N., Gummidi L., Maddila S., Gangu K.K., Jonnalagadda S.B. (2020). A Review on Recent Advances in Nitrogen-Containing Molecules and Their Biological Applications. Molecules.

[14] Taylor A.P., Robinson R.P., Fobian Y.M., Blakemore D.C., Jones L.H., Fadeyi O. (2016). Modern Advances in Heterocyclic Chemistry in Drug Discovery. Org. Biomol. Chem..

[15] Petrou A., Fesatidou M., Geronikaki A. (2021). Thiazole Ring-A Biologically Active Scaffold. Mol. Basel Switz..

[16] Scott K.A., Njardarson J.T. (2018). Analysis of US FDA-Approved Drugs Containing Sulfur Atoms. Top. Curr. Chem..

[17] Sharma P.C., Bansal K.K., Sharma A., Sharma D., Deep A. (2020). Thiazole-Containing Compounds as Therapeutic Targets for Cancer Therapy. Eur. J. Med. Chem..

[18] Pola S. (2016). Significance of Thiazole-Based Heterocycles for Bioactive Systems.

[19] Ali S.H., Sayed A.R. (2021). Review of the Synthesis and Biological Activity of Thiazoles. Synth. Commun..

[20] Makam P., Thakur P.K., Kannan T. (2014). In Vitro and in Silico Antimalarial Activity of 2-(2-Hydrazinyl)Thiazole Derivatives. Eur. J. Pharm. Sci..

[21] Jadav S.S., Kaptein S., Timiri A., De Burghgraeve T., Badavath V.N., Ganesan R., Sinha B.N., Neyts J., Leyssen P., Jayaprakash V. (2015). Design, Synthesis, Optimization and Antiviral Activity of a Class of Hybrid Dengue Virus E Protein Inhibitors. Bioorg. Med. Chem. Lett..

[22] Alsharekh M.M., Althagafi I.I., Shaaban M.R., Farghaly T.A. (2019). Microwave-Assisted and Thermal Synthesis of Nanosized Thiazolyl-Phenothiazine Derivatives and Their Biological Activities. Res. Chem. Intermed..

[23] Yurttaş L., Çavuşoğlu B.K., Cantürk Z. (2020). Novel 2-(2-Hydrazinyl)Thiazole Derivatives as Chemotherapeutic Agents. Synth. Commun..

[24] de Santana T.I., Barbosa M.D.O., Gomes P.A.T.D.M., da Cruz A.C.N., da Silva T.G., Leite A.C.L. (2018). Synthesis, Anticancer Activity and Mechanism of Action of New Thiazole Derivatives. Eur. J. Med. Chem..

[25] Filimonov A.S., Chepanova A.A., Luzina O.A., Zakharenko A.L., Zakharova O.D., Ilina E.S., Dyrkheeva N.S., Kuprushkin M.S., Kolotaev A.V., Khachatryan D.S. (2019). New Hydrazinothiazole Derivatives of Usnic Acid as Potent Tdp1 Inhibitors. Molecules.

[26] Zakharenko A.L., Luzina O.A., Sokolov D.N., Kaledin V.I., Nikolin V.P., Popova N.A., Patel J., Zakharova O.D., Chepanova A.A., Zafar A. (2019). Novel Tyrosyl-DNA Phosphodiesterase 1 Inhibitors Enhance the Therapeutic Impact of Topotecan on in Vivo Tumor Models. Eur. J. Med. Chem..

[27] Chimenti F., Bizzarri B., Maccioni E., Secci D., Bolasco A., Chimenti P., Fioravanti R., Granese A., Carradori S., Tosi F. (2009). A Novel Histone Acetyltransferase Inhibitor Modulating Gcn5 Network: Cyclopentylidene-[4-(4′-Chlorophenyl)Thiazol-2-Yl)Hydrazone. J. Med. Chem..

[28] Secci D., Carradori S., Bizzarri B., Bolasco A., Ballario P., Patramani Z., Fragapane P., Vernarecci S., Canzonetta C., Filetici P. (2014). Synthesis of a Novel Series of Thiazole-Based Histone Acetyltransferase Inhibitors. Bioorg. Med. Chem..

[29] Yan J.-D., Liu Y., Zhang Z.-Y., Liu G.-Y., Xu J.-H., Liu L.-Y., Hu Y.-M. (2015). Expression and Prognostic Significance of VEGFR-2 in Breast Cancer. Pathol.-Res. Pract..

[30] Ghosh S., Sullivan C.A.W., Zerkowski M.P., Molinaro A.M., Rimm D.L., Camp R.L., Chung G.G. (2008). High Levels of Vascular Endothelial Growth Factor and Its Receptors (VEGFR-1, VEGFR-2, Neuropilin-1) Are Associated with Worse Outcome in Breast Cancer. Hum. Pathol..

[31] Miyoshi Y., Murase K., Saito M., Imamura M., Oh K. (2010). Mechanisms of Estrogen Receptor-α Upregulation in Breast Cancers. Med. Mol. Morphol..

[32] Hua H., Zhang H., Kong Q., Jiang Y. (2018). Mechanisms for Estrogen Receptor Expression in Human Cancer. Exp. Hematol. Oncol..

[33] Zhang J., Liu C., Shi W., Yang L., Zhang Q., Cui J., Fang Y., Li Y., Ren G., Yang S. (2016). The Novel VEGF Receptor 2 Inhibitor YLL545 Inhibits Angiogenesis and Growth in Breast Cancer. Oncotarget.

[34] Ni H., Guo M., Zhang X., Jiang L., Tan S., Yuan J., Cui H., Min Y., Zhang J., Schlisio S. (2021). VEGFR2 Inhibition Hampers Breast Cancer Cell Proliferation via Enhanced Mitochondrial Biogenesis. Cancer Biol. Med..

[35] Gaber A., Refat M., Belal A., El-Deen I., Hassan N., Zakaria R., Alhomrani M., Alamri A., Alsanie W., Saied E.M. (2021). New Mononuclear and Binuclear Cu(II), Co(II), Ni(II), and Zn(II) Thiosemicarbazone Complexes with Potential Biological Activity: Antimicrobial and Molecular Docking Study. Molecules.

[36] Gaber A., Alsanie W.F., Kumar D.N., Refat M.S., Saied E.M. (2020). Novel Papaverine Metal Complexes with Potential Anticancer Activities. Molecules.

[37] Saied E.M., Banhart S., Bürkle S.E., Heuer D., Arenz C. (2015). A Series of Ceramide Analogs Modified at the 1-Position with Potent Activity against the Intracellular Growth of Chlamydia Trachomatis. Future Med. Chem..

[38] Banhart S., Saied E.M., Martini A., Koch S., Aeberhard L., Madela K., Arenz C., Heuer D. (2014). Improved Plaque Assay Identifies a Novel Anti-Chlamydia Ceramide Derivative with Altered Intracellular Localization. Antimicrob. Agents Chemother..

[39] Saied E.M., Diederich S., Arenz C. (2014). Facile Synthesis of the CERT Inhibitor HPA-12 and Some Novel Derivatives. Chem.-Asian J..

[40] Refat M.S., Ibrahim H.K., Sowellim S.Z.A., Soliman M.H., Saeed E.M. (2009). Spectroscopic and Thermal Studies of Mn(II), Fe(III), Cr(III) and Zn(II) Complexes Derived from the Ligand Resulted by the Reaction Between 4-Acetyl Pyridine and Thiosemicarbazide. J. Inorg. Organomet. Polym. Mater..

[41] Saied E.M., Le T.L.-S., Hornemann T., Arenz C. (2018). Synthesis and Characterization of Some Atypical Sphingoid Bases. Bioorg. Med. Chem..

[42] Saied E.M., Arenz C. (2021). Stereoselective Synthesis of Novel Sphingoid Bases Utilized for Exploring the Secrets of Sphinx. Int. J. Mol. Sci..

[43] El Azab I.H., Saied E.M., Osman A.A., Mehana A.E., Saad H.A., Elkanzi N.A. (2021). Novel N-Bridged Pyrazole-1-Carbothioamides with Potential Antiproliferative Activity: Design, Synthesis, in Vitro and in Silico Studies. Future Med. Chem..

[44] Ignat A., Lovasz T., Vasilescu M., Fischer-Fodor E., Tatomir C.B., Cristea C., Silaghi-Dumitrescu L., Zaharia V. (2012). Heterocycles 27. Microwave Assisted Synthesis and Antitumour Activity of Novel Phenothiazinyl-Thiazolyl-Hydrazine Derivatives. Arch. Pharm..

[45] Zaharia V., Ignat A., Palibroda N., Ngameni B., Kuete V., Fokunang C.N., Moungang M.L., Ngadjui B.T. (2010). Synthesis of Some P-Toluenesulfonyl-Hydrazinothiazoles and Hydrazino-Bis-Thiazoles and Their Anticancer Activity. Eur. J. Med. Chem..

[46] Zhang D.-N., Li J.-T., Song Y.-L., Liu H.-M., Li H.-Y. (2012). Efficient One-Pot Three-Component Synthesis of N-(4-Arylthiazol-2-Yl) Hydrazones in Water under Ultrasound Irradiation. Ultrason. Sonochem..

[47] Borcea A.-M., Ionuț I., Crișan O., Oniga O. (2021). An Overview of the Synthesis and Antimicrobial, Antiprotozoal, and Antitumor Activity of Thiazole and Bisthiazole Derivatives. Molecules.

[48] Grozav A., Găină L.I., Pileczki V., Crisan O., Silaghi-Dumitrescu L., Therrien B., Zaharia V., Berindan-Neagoe I. (2014). The Synthesis and Antiproliferative Activities of New Arylidene-Hydrazinyl-Thiazole Derivatives. Int. J. Mol. Sci..

[49] Guerrero-Pepinosa N.Y., Cardona-Trujillo M.C., Garzón-Castaño S.C., Veloza L.A., Sepúlveda-Arias J.C. (2021). Antiproliferative Activity of Thiazole and Oxazole Derivatives: A Systematic Review of in vitro and in vivo Studies. Biomed. Pharmacother..

[50] Herrera-España A.D., Us-Martín J., Quintal-Novelo C., Mirón-López G., Quijano-Quiñones R.F., Cáceres-Castillo D., Graniel-Sabido M., Moo-Puc R.E., Mena-Rejón G.J. (2021). Cytotoxic and Antiproliferative Activity of Thiazole Derivatives of Ochraceolide A. Nat. Prod. Res..

[51] Sun M., Xu Q., Xu J., Wu Y., Wang Y., Zuo D., Guan Q., Bao K., Wang J., Wu Y. (2017). Synthesis and Bioevaluation of N,4-Diaryl-1,3-Thiazole-2-Amines as Tubulin Inhibitors with Potent Antiproliferative Activity. PLoS ONE.

[52] Bimoussa A., Oubella A., El Mansouri A., Fawzi M., Laamari Y., Auhmani A., Itto M.Y.A., Morjani H., Auhmani A. (2022). Synthesis and Biological Evaluation of Novel Thiazole Analogs with Both Anti-Proliferative and Mechanistic Analyses and Molecular Docking Studies. ChemistrySelect.

[53] Ramos-Inza S., Aydillo C., Sanmartín C., Plano D. (2019). Thiazole Moiety: An Interesting Scaffold for Developing New Antitumoral Compounds.

[54] Senger D.R., Galli S.J., Dvorak A.M., Perruzzi C.A., Harvey V.S., Dvorak H.F. (1983). Tumor Cells Secrete a Vascular Permeability Factor That Promotes Accumulation of Ascites Fluid. Science.

[55] Olsson A.-K., Dimberg A., Kreuger J., Claesson-Welsh L. (2006). VEGF Receptor Signalling—In Control of Vascular Function. Nat. Rev. Mol. Cell Biol..

[56] Guo S., Colbert L.S., Fuller M., Zhang Y., Gonzalez-Perez R.R. (2010). Vascular Endothelial Growth Factor Receptor-2 in Breast Cancer. Biochim. Biophys. Acta.

[57] Higgins K.J., Liu S., Abdelrahim M., Yoon K., Vanderlaag K., Porter W., Metz R.P., Safe S. (2006). Vascular Endothelial Growth Factor Receptor-2 Expression Is Induced by 17β-Estradiol in ZR-75 Breast Cancer Cells by Estrogen Receptor α/Sp Proteins. Endocrinology.

[58] Peng F.-W., Liu D.-K., Zhang Q.-W., Xu Y.-G., Shi L. (2017). VEGFR-2 Inhibitors and the Therapeutic Applications Thereof: A Patent Review (2012-2016). Expert Opin. Ther. Pat..

[59] Alanazi M.M., Eissa I.H., Alsaif N.A., Obaidullah A.J., Alanazi W.A., Alasmari A.F., Albassam H., Elkady H., Elwan A. (2021). Design, Synthesis, Docking, ADMET Studies, and Anticancer Evaluation of New 3-Methylquinoxaline Derivatives as VEGFR-2 Inhibitors and Apoptosis Inducers. J. Enzym. Inhib. Med. Chem..

[60] Alanazi M.M., Elwan A., Alsaif N.A., Obaidullah A.J., Alkahtani H.M., Al-Mehizia A.A., Alsubaie S.M., Taghour M.S., Eissa I.H. (2021). Discovery of New 3-Methylquinoxalines as Potential Anti-Cancer Agents and Apoptosis Inducers Targeting VEGFR-2: Design, Synthesis, and in Silico Studies. J. Enzym. Inhib. Med. Chem..

[61] Alsaif N.A., Taghour M.S., Alanazi M.M., Obaidullah A.J., Al-Mehizia A.A., Alanazi M.M., Aldawas S., Elwan A., Elkady H. (2021). Discovery of New VEGFR-2 Inhibitors Based on Bis([1, 2, 4]Triazolo)[4,3-a:3’,4’-c]Quinoxaline Derivatives as Anticancer Agents and Apoptosis Inducers. J. Enzym. Inhib. Med. Chem..

[62] Ghorab M.M., Alsaid M.S., Soliman A.M., Ragab F.A. (2017). VEGFR-2 Inhibitors and Apoptosis Inducers: Synthesis and Molecular Design of New Benzo[g]Quinazolin Bearing Benzenesulfonamide Moiety. J. Enzym. Inhib. Med. Chem..

[63] El-Naggar A.M., Zidan A., Elkaeed E.B., Taghour M.S., Badawi W.A. (2022). Design, Synthesis and Docking Studies of New Hydrazinyl-Thiazole Derivatives as Anticancer and Antimicrobial Agents. J. Saudi Chem. Soc..

[64] Ayati A., Emami S., Moghimi S., Foroumadi A. (2019). Thiazole in the Targeted Anticancer Drug Discovery. Future Med. Chem..

[65] Wang G., Liu W., Fan M., He M., Li Y., Peng Z. (2021). Design, Synthesis and Biological Evaluation of Novel Thiazole-Naphthalene Derivatives as Potential Anticancer Agents and Tubulin Polymerisation Inhibitors. J. Enzym. Inhib. Med. Chem..

[66] Iwata H., Oki H., Okada K., Takagi T., Tawada M., Miyazaki Y., Imamura S., Hori A., Lawson J.D., Hixon M.S. (2012). A Back-to-Front Fragment-Based Drug Design Search Strategy Targeting the DFG-Out Pocket of Protein Tyrosine Kinases. ACS Med. Chem. Lett..

[67] McTigue M., Murray B.W., Chen J.H., Deng Y.-L., Solowiej J., Kania R.S. (2012). Molecular Conformations, Interactions, and Properties Associated with Drug Efficiency and Clinical Performance among VEGFR TK Inhibitors. Proc. Natl. Acad. Sci. USA.

[68] McAulay K., Hoyt E.A., Thomas M., Schimpl M., Bodnarchuk M.S., Lewis H.J., Barratt D., Bhavsar D., Robinson D.M., Deery M.J. (2020). Alkynyl Benzoxazines and Dihydroquinazolines as Cysteine Targeting Covalent Warheads and Their Application in Identification of Selective Irreversible Kinase Inhibitors. J. Am. Chem. Soc..

[69] Kettle J.G., Anjum R., Barry E., Bhavsar D., Brown C., Boyd S., Campbell A., Goldberg K., Grondine M., Guichard S. (2018). Discovery of N-(4-{[5-Fluoro-7-(2-Methoxyethoxy)Quinazolin-4-Yl]Amino}phenyl)-2-[4-(Propan-2-Yl)-1H-1,2,3-Triazol-1-Yl]Acetamide (AZD3229), a Potent Pan-KIT Mutant Inhibitor for the Treatment of Gastrointestinal Stromal Tumors. J. Med. Chem..

[70] Norman M.H., Liu L., Lee M., Xi N., Fellows I., D’Angelo N.D., Dominguez C., Rex K., Bellon S.F., Kim T.-S. (2012). Structure-Based Design of Novel Class II c-Met Inhibitors: 1. Identification of Pyrazolone-Based Derivatives. J. Med. Chem..

[71] Hasegawa M., Nishigaki N., Washio Y., Kano K., Harris P.A., Sato H., Mori I., West R.I., Shibahara M., Toyoda H. (2007). Discovery of Novel Benzimidazoles as Potent Inhibitors of TIE-2 and VEGFR-2 Tyrosine Kinase Receptors. J. Med. Chem..

[72] Hilberg F., Roth G.J., Krssak M., Kautschitsch S., Sommergruber W., Tontsch-Grunt U., Garin-Chesa P., Bader G., Zoephel A., Quant J. (2008). BIBF 1120: Triple Angiokinase Inhibitor with Sustained Receptor Blockade and Good Antitumor Efficacy. Cancer Res..

[73] Harris P.A., Cheung M., Hunter R.N., Brown M.L., Veal J.M., Nolte R.T., Wang L., Liu W., Crosby R.M., Johnson J.H. (2005). Discovery and Evaluation of 2-Anilino-5-Aryloxazoles as a Novel Class of VEGFR2 Kinase Inhibitors. J. Med. Chem..

[74] Miyazaki Y., Matsunaga S., Tang J., Maeda Y., Nakano M., Philippe R.J., Shibahara M., Liu W., Sato H., Wang L. (2005). Novel 4-Amino-Furo[2,3-d]Pyrimidines as Tie-2 and VEGFR2 Dual Inhibitors. Bioorg. Med. Chem. Lett..

[75] Oguro Y., Miyamoto N., Okada K., Takagi T., Iwata H., Awazu Y., Miki H., Hori A., Kamiyama K., Imamura S. (2010). Design, Synthesis, and Evaluation of 5-Methyl-4-Phenoxy-5H-Pyrrolo[3,2-d]Pyrimidine Derivatives: Novel VEGFR2 Kinase Inhibitors Binding to Inactive Kinase Conformation. Bioorg. Med. Chem..

[76] Okaniwa M., Hirose M., Imada T., Ohashi T., Hayashi Y., Miyazaki T., Arita T., Yabuki M., Kakoi K., Kato J. (2012). Design and Synthesis of Novel DFG-Out RAF/Vascular Endothelial Growth Factor Receptor 2 (VEGFR2) Inhibitors. 1. Exploration of [5,6]-Fused Bicyclic Scaffolds. J. Med. Chem..

[77] Bold G., Schnell C., Furet P., McSheehy P., Brüggen J., Mestan J., Manley P.W., Drückes P., Burglin M., Dürler U. (2016). A Novel Potent Oral Series of VEGFR2 Inhibitors Abrogate Tumor Growth by Inhibiting Angiogenesis. J. Med. Chem..

[78] Patterson J.R., Graves A.P., Stoy P., Cheung M., Desai T.A., Fries H., Gatto G.J., Holt D.A., Shewchuk L., Totoritis R. (2021). Identification of Diarylurea Inhibitors of the Cardiac-Specific Kinase TNNI3K by Designing Selectivity Against VEGFR2, P38α, and B-Raf. J. Med. Chem..

[79] Mosmann T. (1983). Rapid Colorimetric Assay for Cellular Growth and Survival: Application to Proliferation and Cytotoxicity Assays. J. Immunol. Methods.

[80] Denizot F., Lang R. (1986). Rapid Colorimetric Assay for Cell Growth and Survival: Modifications to the Tetrazolium Dye Procedure Giving Improved Sensitivity and Reliability. J. Immunol. Methods.

[81] Abou-Seri S.M., Eldehna W.M., Ali M.M., Abou El Ella D.A. (2016). 1-Piperazinylphthalazines as Potential VEGFR-2 Inhibitors and Anticancer Agents: Synthesis and in Vitro Biological Evaluation. Eur. J. Med. Chem..

[82] Amin N.H., Elsaadi M.T., Zaki S.S., Abdel-Rahman H.M. (2020). Design, Synthesis and Molecular Modeling Studies of 2-Styrylquinazoline Derivatives as EGFR Inhibitors and Apoptosis Inducers. Bioorganic Chem..

[83] Kuroda K., Fukuda T., Isogai H., Okumura K., Krstic-Demonacos M., Isogai E. (2015). Antimicrobial Peptide FF/CAP18 Induces Apoptotic Cell Death in HCT116 Colon Cancer Cells via Changes in the Metabolic Profile. Int. J. Oncol..

[84] Abu Almaaty A.H., Elgrahy N.A., Fayad E., Abu Ali O.A., Mahdy A.R.E., Barakat L.A.A., El Behery M. (2021). Design, Synthesis and Anticancer Evaluation of Substituted Cinnamic Acid Bearing 2-Quinolone Hybrid Derivatives. Molecules.

[85] Mohammed F.Z., Rizzk Y.W., El-Deen I.M., Gad E.M., El Behery M., Mahdy A.R.E. (2022). Discovery of 2-Amino-4H-1, 3, 4-Thiadiazine-5(6H)-One Derivatives and Their In Vitro Antitumor Investigation. ChemistrySelect.

[86] Saied E.M., El-Maradny Y.A., Osman A.A., Darwish A.M.G., Abo Nahas H.H., Niedbała G., Piekutowska M., Abdel-Rahman M.A., Balbool B.A., Abdel-Azeem A.M. (2021). A Comprehensive Review about the Molecular Structure of Severe Acute Respiratory Syndrome Coronavirus 2 (SARS-CoV-2): Insights into Natural Products against COVID-19. Pharmaceutics.

[87] Healey R.D., Saied E.M., Cong X., Karsai G., Gabellier L., Saint-Paul J., Del Nero E., Jeannot S., Drapeau M., Fontanel S. (2022). Discovery and Mechanism of Action of Small Molecule Inhibitors of Ceramidases**. Angew. Chem. Int. Ed..

[88] Mohamed D.I., Alaa El-Din Aly El-Waseef D., Nabih E.S., El-Kharashi O.A., Abd El-Kareem H.F., Abo Nahas H.H., Abdel-Wahab B.A., Helmy Y.A., Alshawwa S.Z., Saied E.M. (2022). Acetylsalicylic Acid Suppresses Alcoholism-Induced Cognitive Impairment Associated with Atorvastatin Intake by Targeting Cerebral MiRNA155 and NLRP3: In Vivo, and In Silico Study. Pharmaceutics.

[89] Samaha D., Hamdo H.H., Cong X., Schumacher F., Banhart S., Aglar Ö., Möller H.M., Heuer D., Kleuser B., Saied E.M. (2020). Liposomal FRET Assay Identifies Potent Drug-Like Inhibitors of the Ceramide Transport Protein (CERT). Chem. Eur. J..

[90] Mohamed D.I., Abou-Bakr D.A., Ezzat S.F., El-Kareem H.F.A., Nahas H.H.A., Saad H.A., Mehana A.E., Saied E.M. (2021). Vitamin D3 Prevents the Deleterious Effects of Testicular Torsion on Testis by Targeting MiRNA-145 and ADAM17: In Silico and In Vivo Study. Pharmaceuticals.

[91] Mohamed D.I., Ezzat S.F., Elayat W.M., El-Kharashi O.A., El-Kareem H.F.A., Nahas H.H.A., Abdel-Wahab B.A., Alshawwa S.Z., Saleh A., Helmy Y.A. (2022). Hepatoprotective Role of Carvedilol against Ischemic Hepatitis Associated with Acute Heart Failure via Targeting MiRNA-17 and Mitochondrial Dynamics-Related Proteins: An In Vivo and In Silico Study. Pharmaceuticals.

