# Supplementary material for: Novel 1,3-Thiazole Analogues with Potent Activity against Breast Cancer: A Design, Synthesis, In Vitro, and In Silico Study
Source: Molecules. 2022 Jul 31;27(15):4898. doi: 10.3390/molecules27154898 (PMC9370021; doi:10.3390/molecules27154898)
Supplement: Supplementary file 1 [file molecules-27-04898-s001.zip › molecules-1803170-supplementary.pdf]

Supplementary Materials

Novel 1,3-Thiazole Analogues with Potent Activity against Breast Cancer: A Design, Synthesis, In Vitro, and In Silico Study

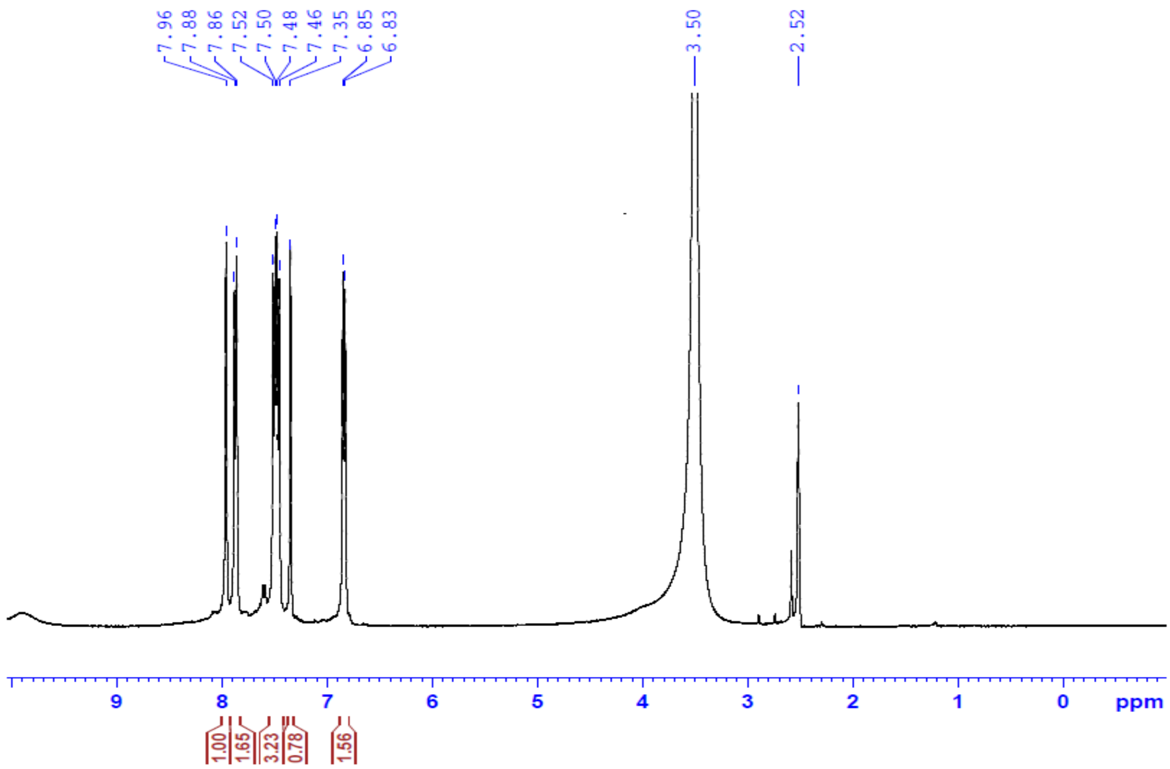

Figure S1a. ^{1}H-NMR spectrum of compound **3a**

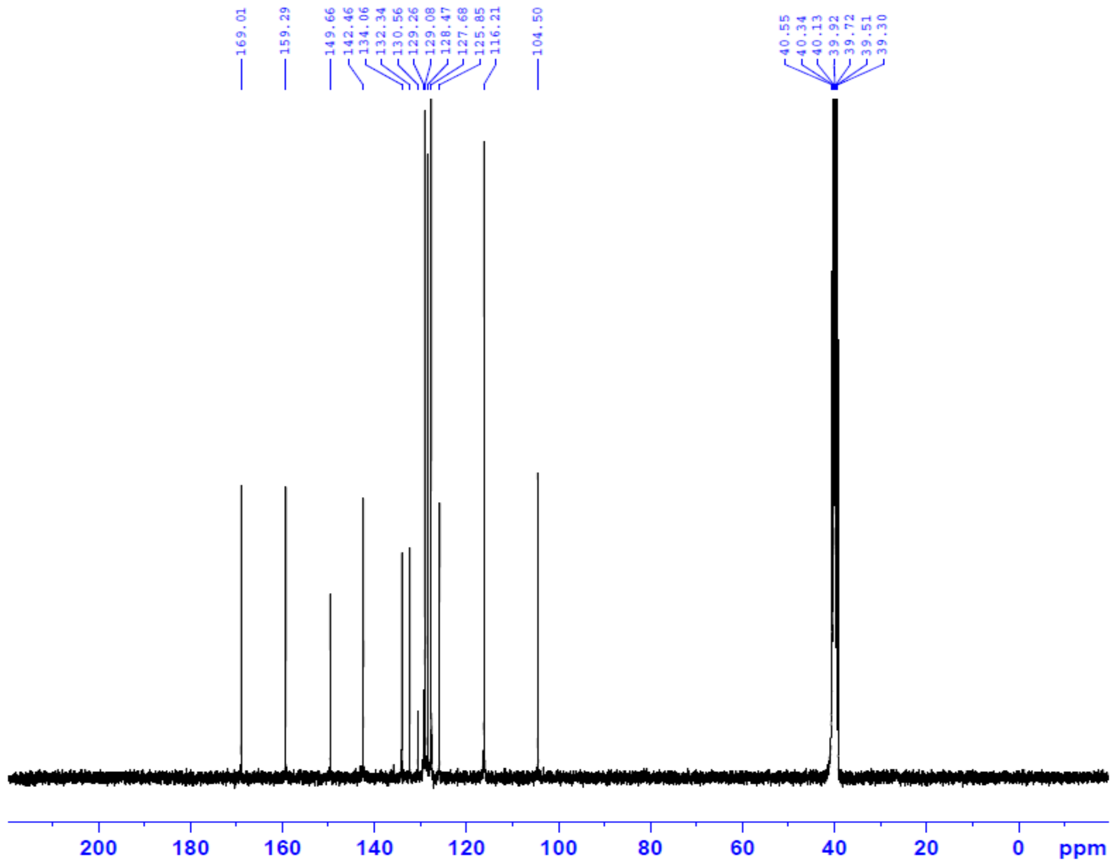

Figure S1b. ^{13}C-NMR spectrum of compound **3a**

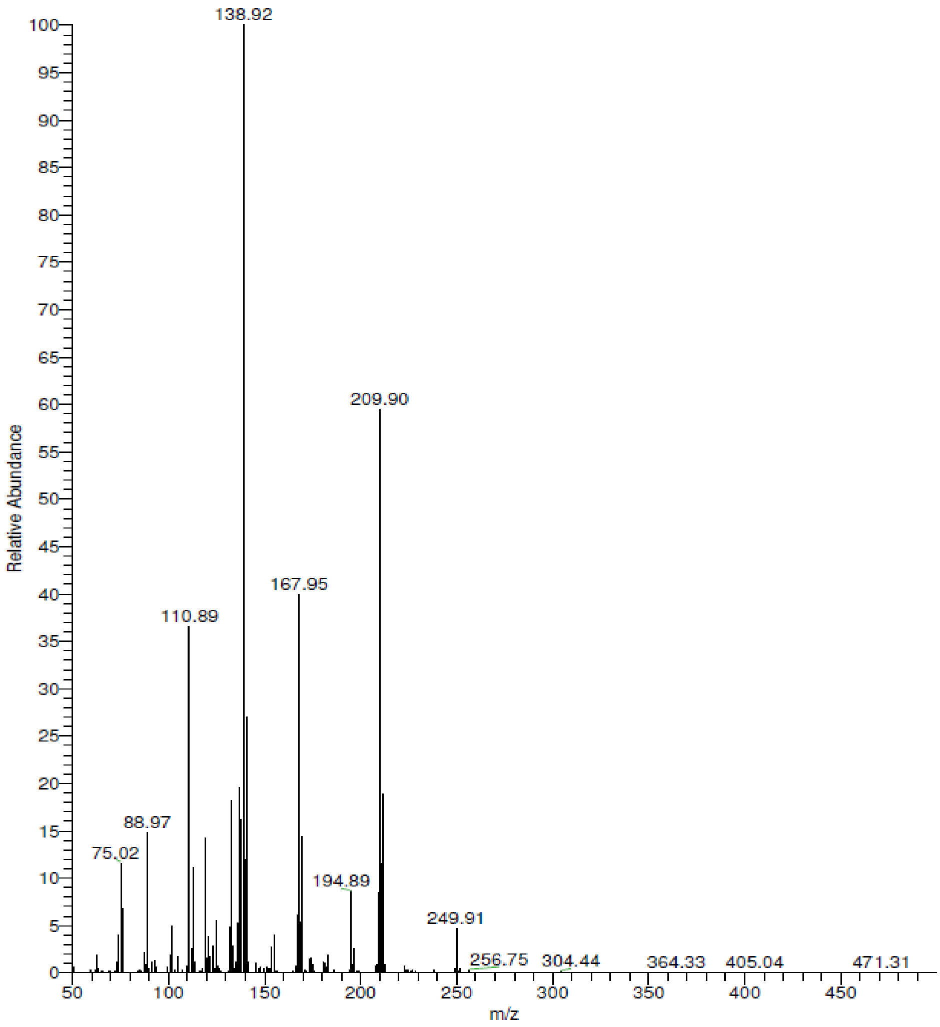

Figure S1c. mass spectrum of compound **3a**

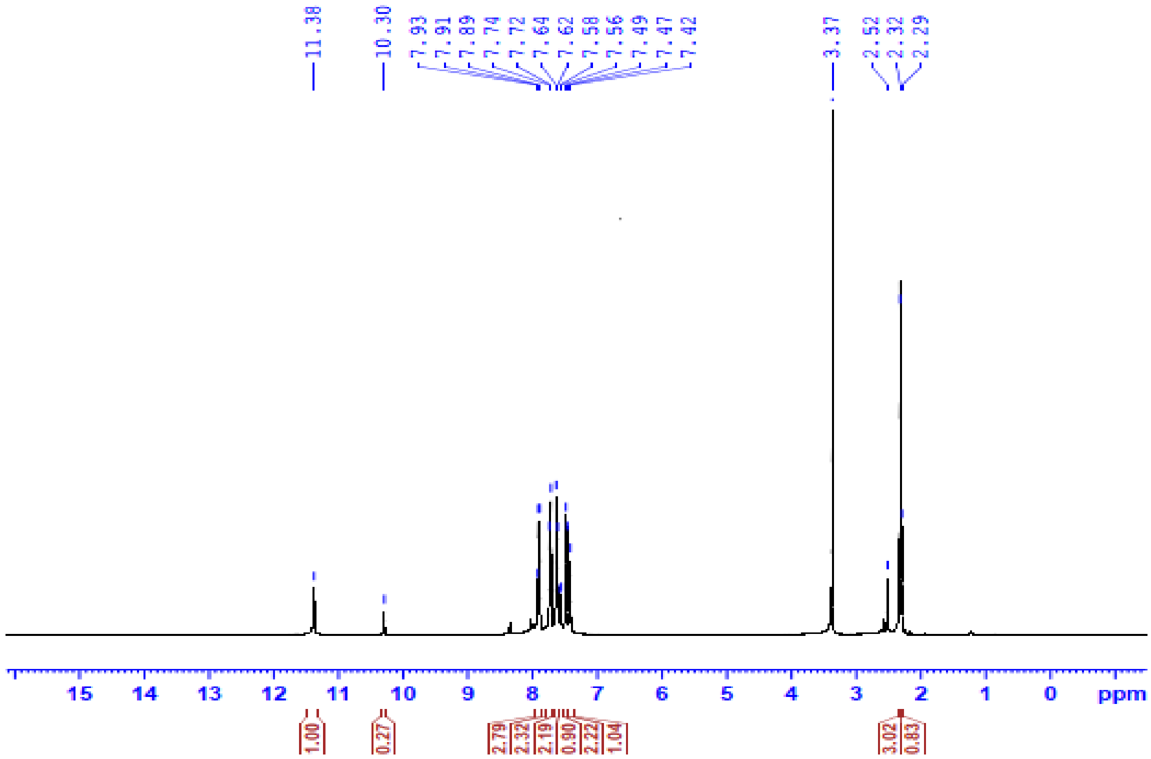

Figure S2a. **^{1}H-NMR spectrum of compound 3b**

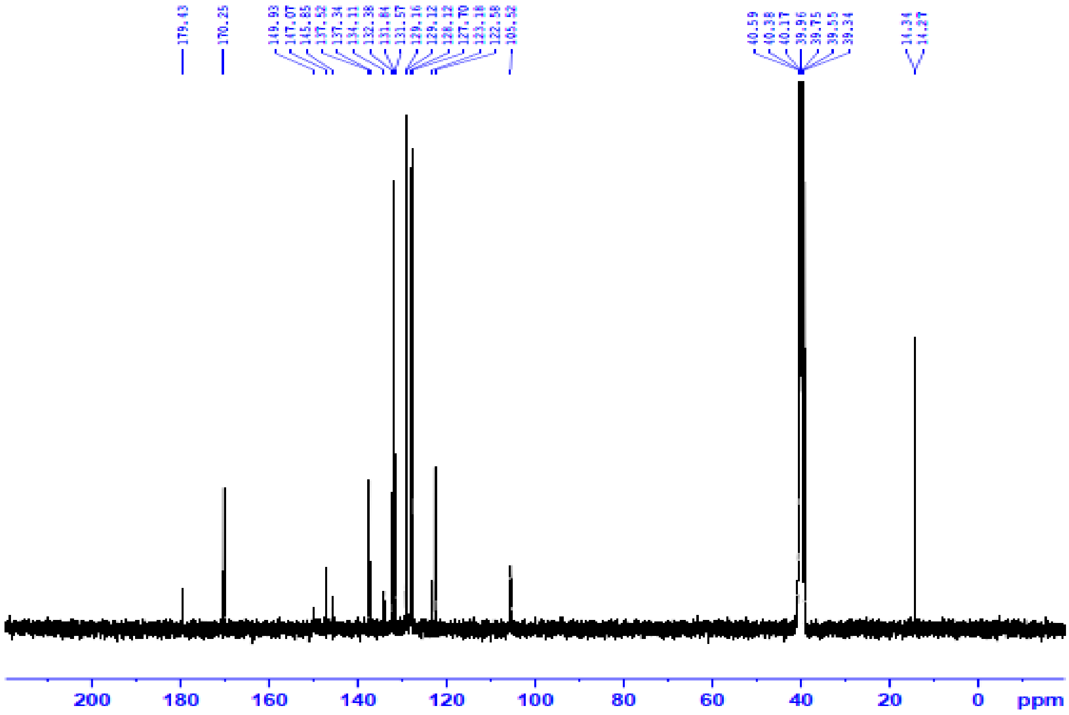

Figure S2b. ^{13}C-NMR spectrum of compound **3b**

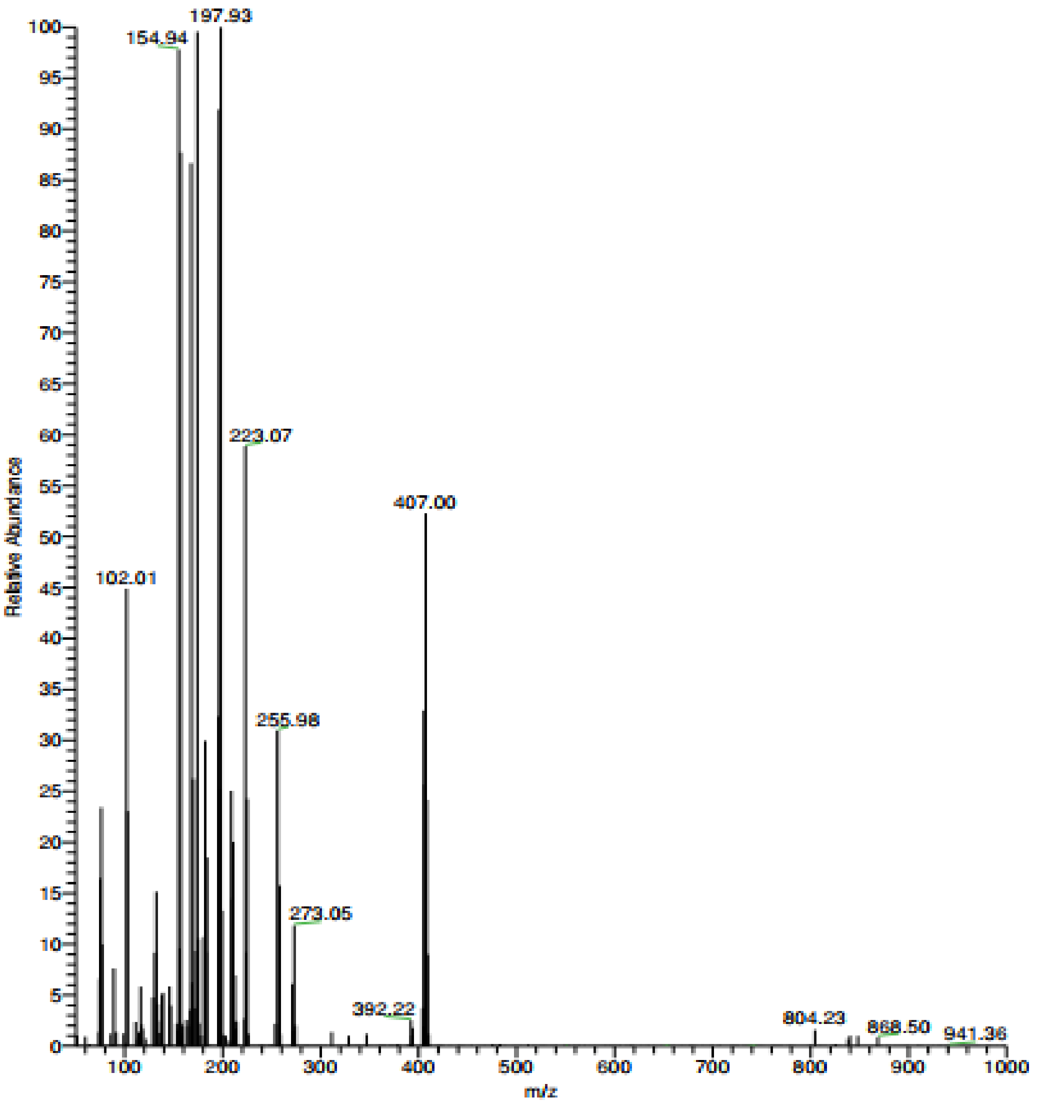

Figure S2c. Mass spectrum of compound **3b**

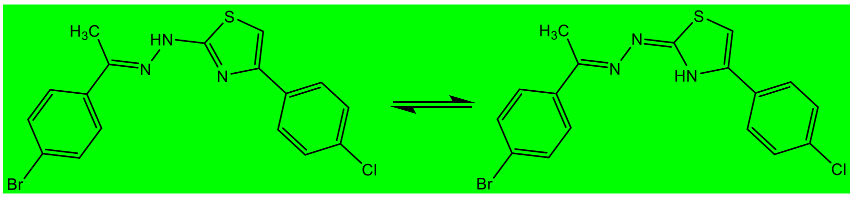

Figure S2d: Thiazole-thiazolidine tautomers of compound **3b**

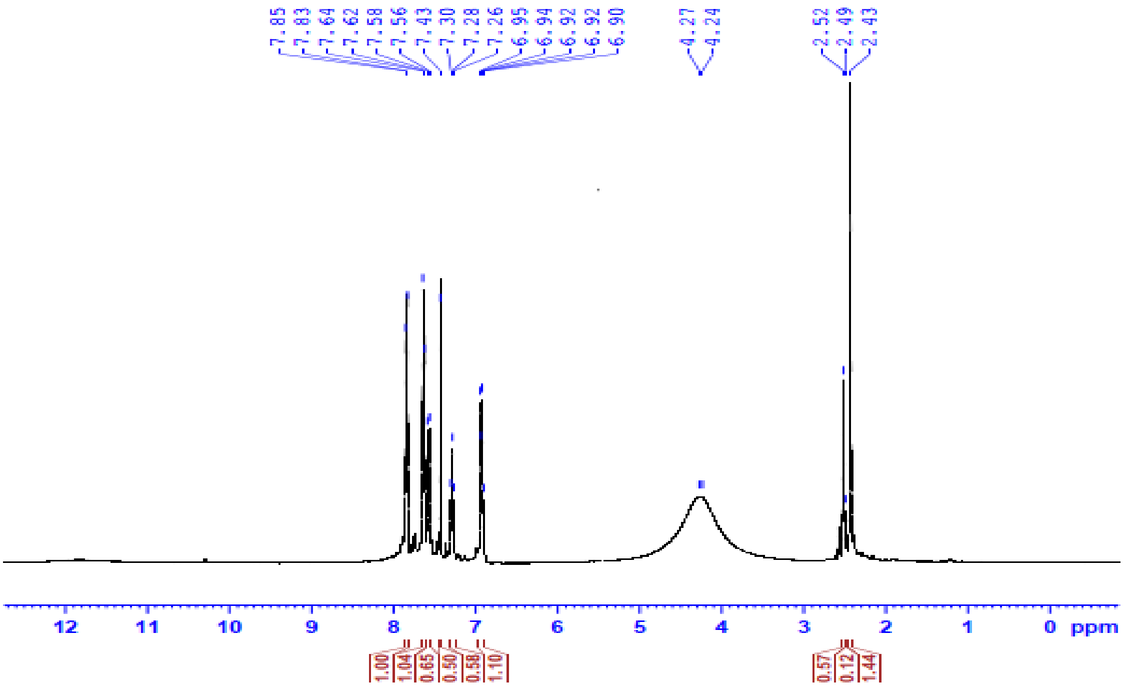

Figure S3a. ^{1}H-NMR spectrum of compound 3c

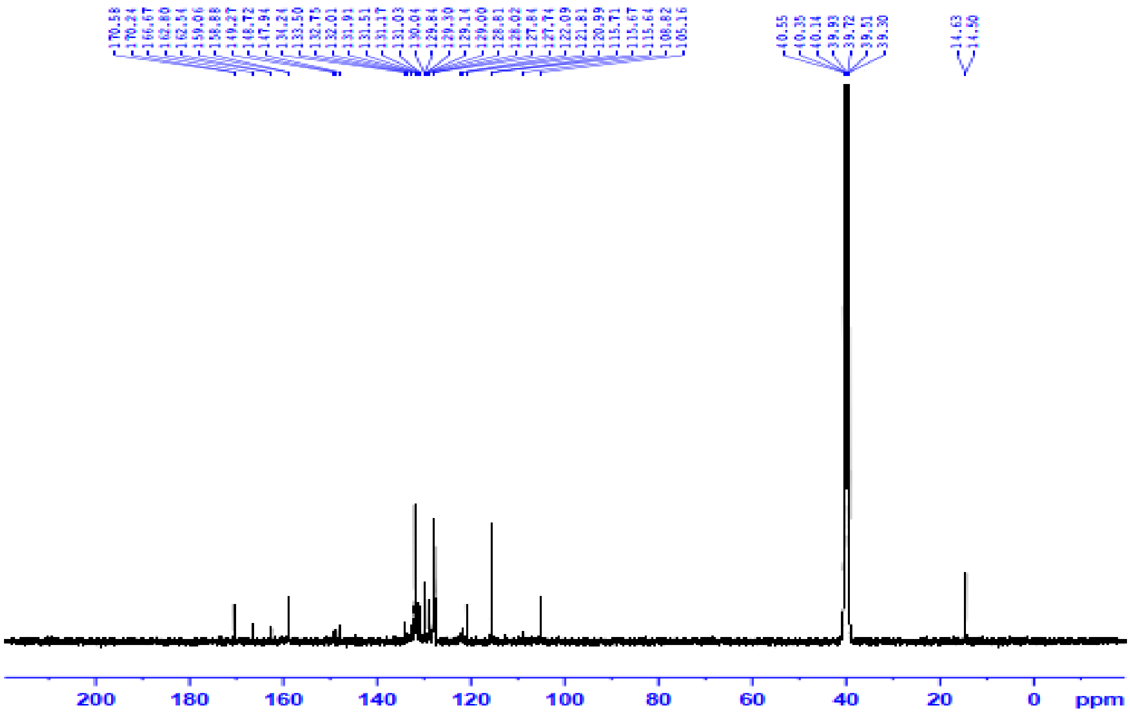

Figure S3b. ^{13}C-NMR spectrum of compound 3c

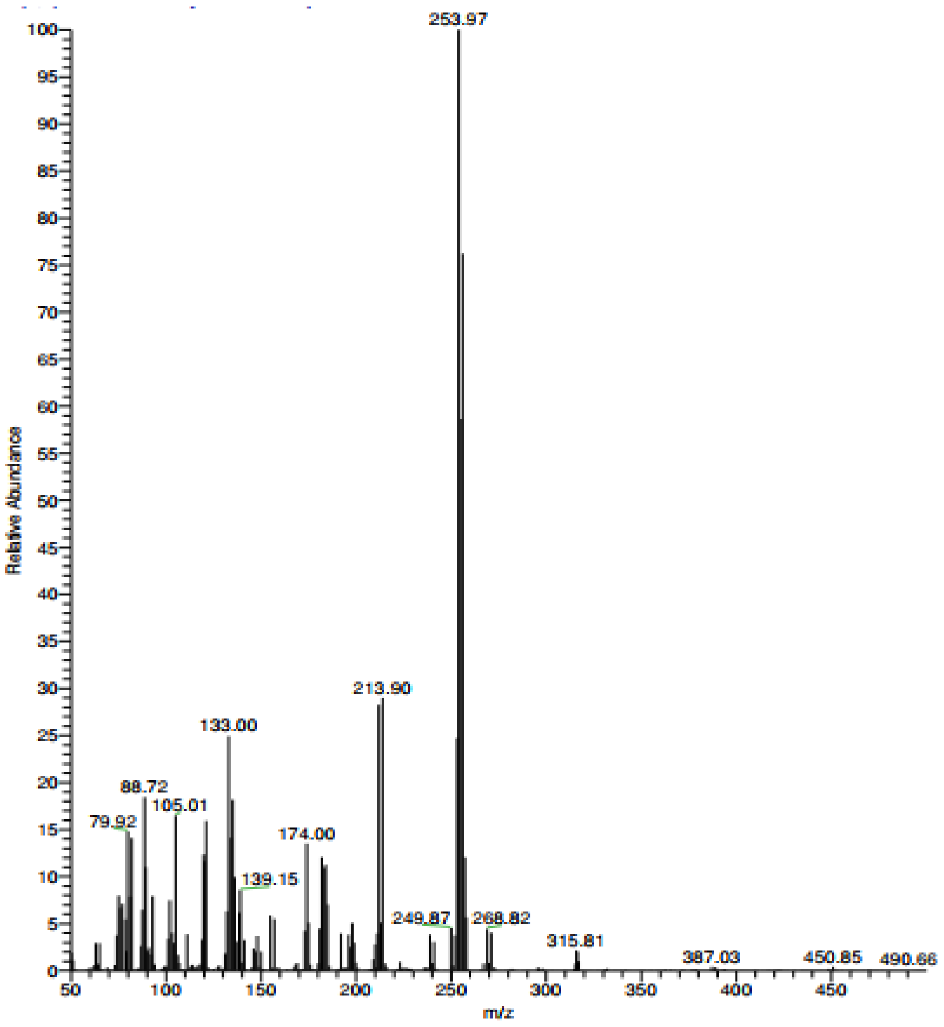

Figure S3c. Mass spectrum of compound **3c**

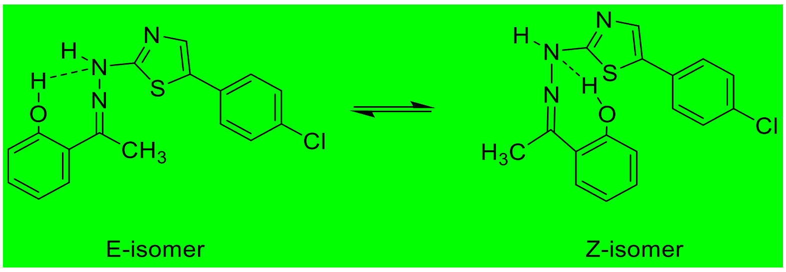

Figure S3d: The *E* and *Z* isomer of compound **3c**, and the formed hydrogen bond between phenolic hydroxyl with NH

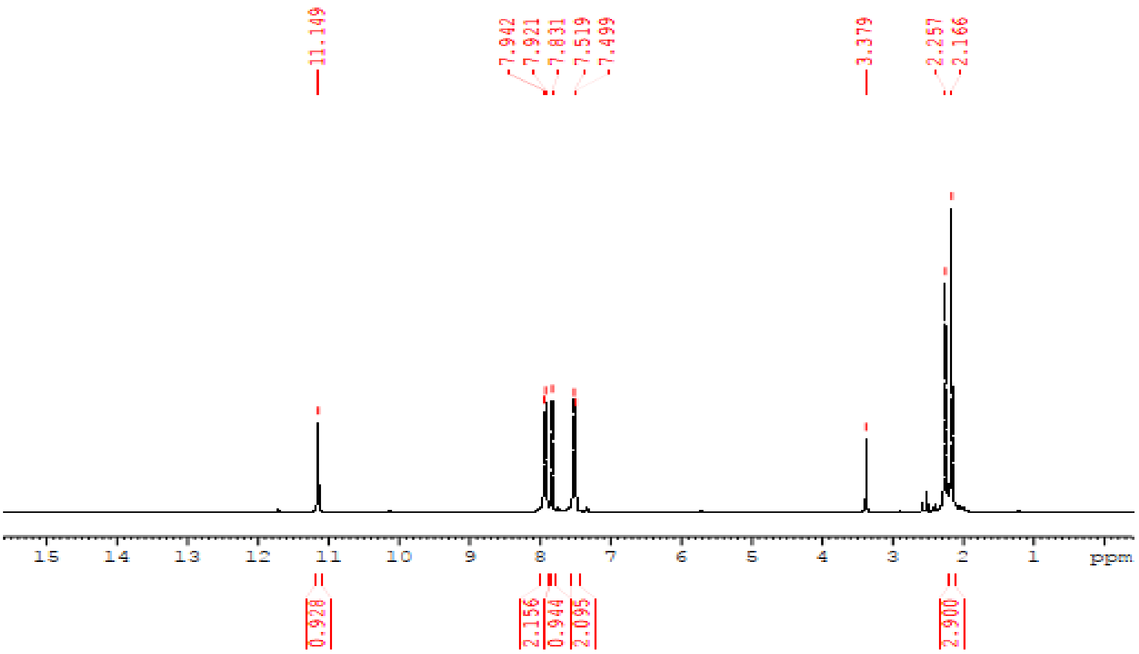

Figure S4a. ^{1}H-NMR spectrum of compound 4

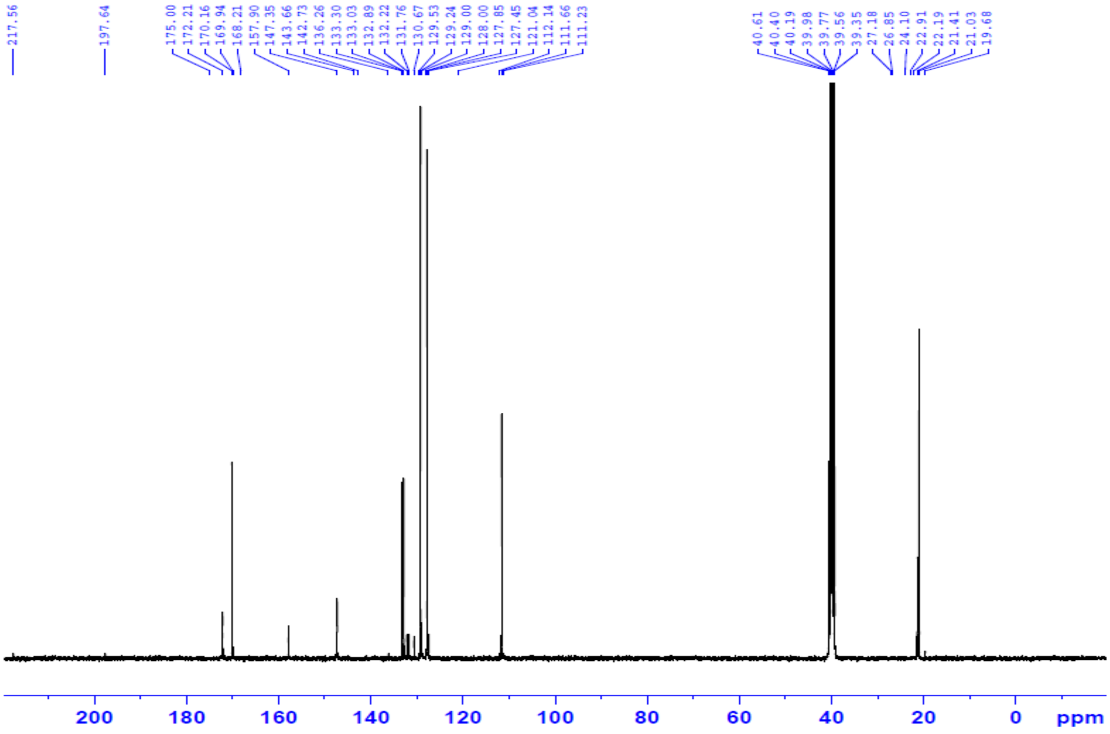

Figure S4b. ^{13}C-NMR spectrum of compound 4

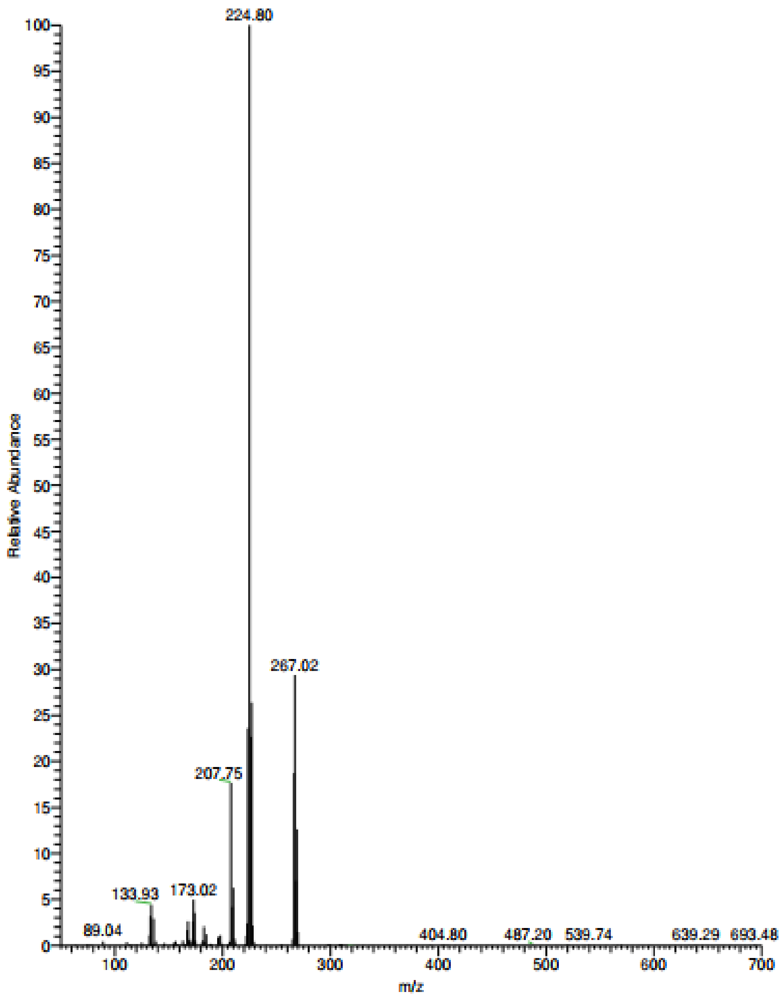

Figure S4c. Mass spectrum of compound **4**

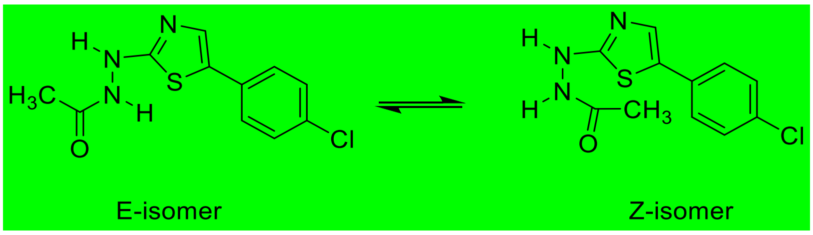

Figure S4d. Two isomers E and Z of compound (**4**)

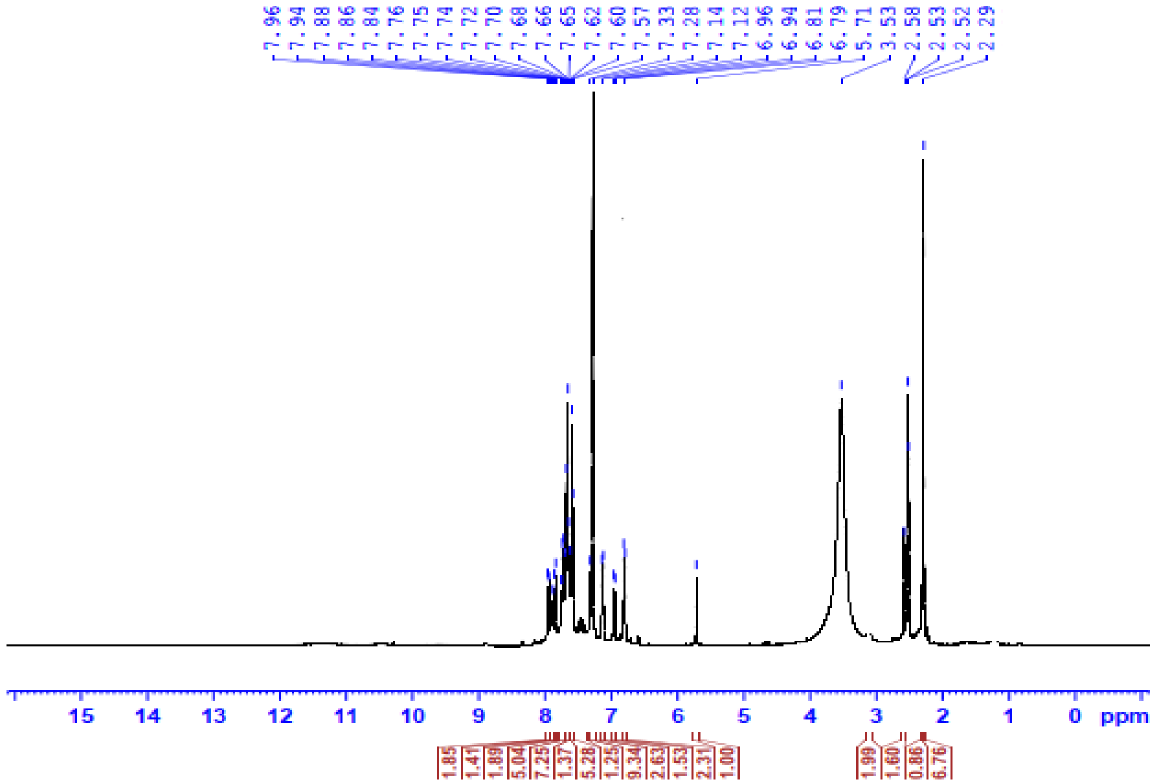

Figure S5a. ^{1}**H-NMR spectrum of compound 5a**

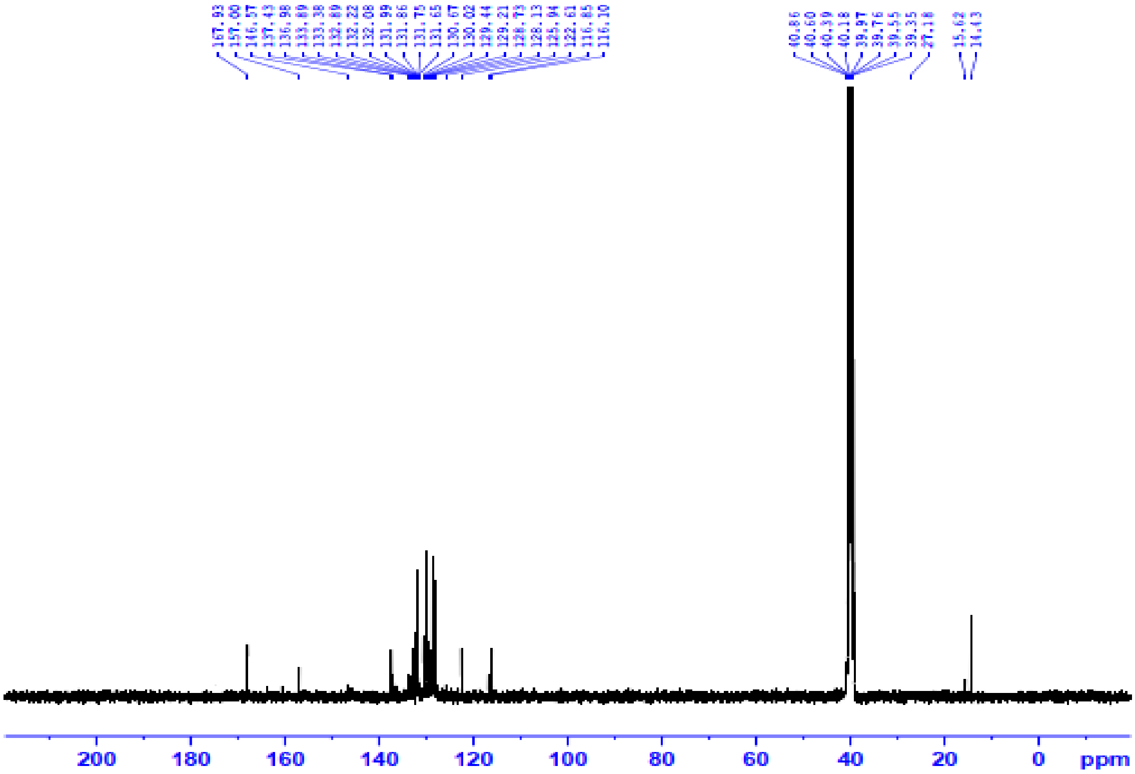

Figure S5b. ^{13}**C-NMR spectrum of compound 5a**

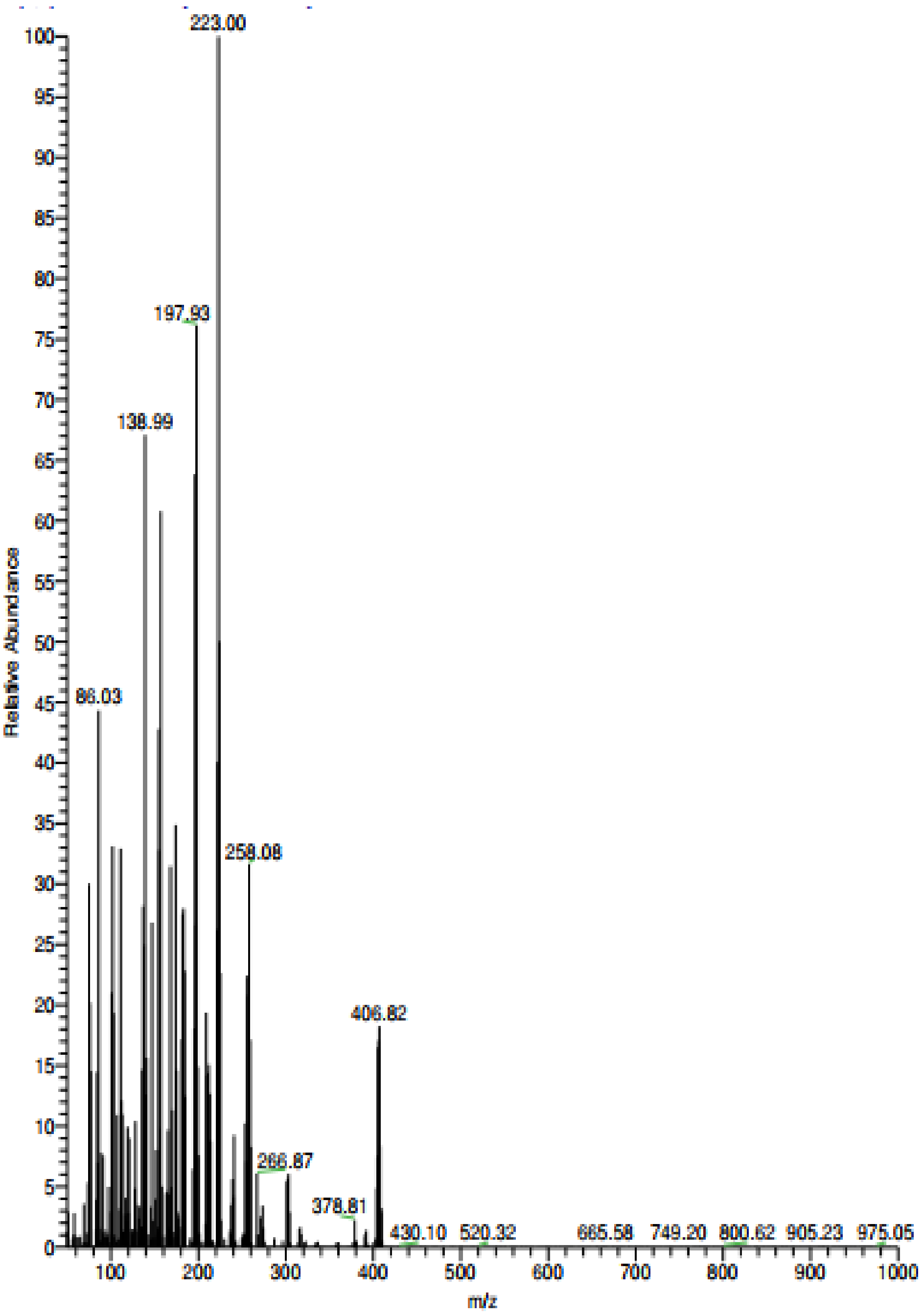

Figure S5c. Mass spectrum of compound **5a**

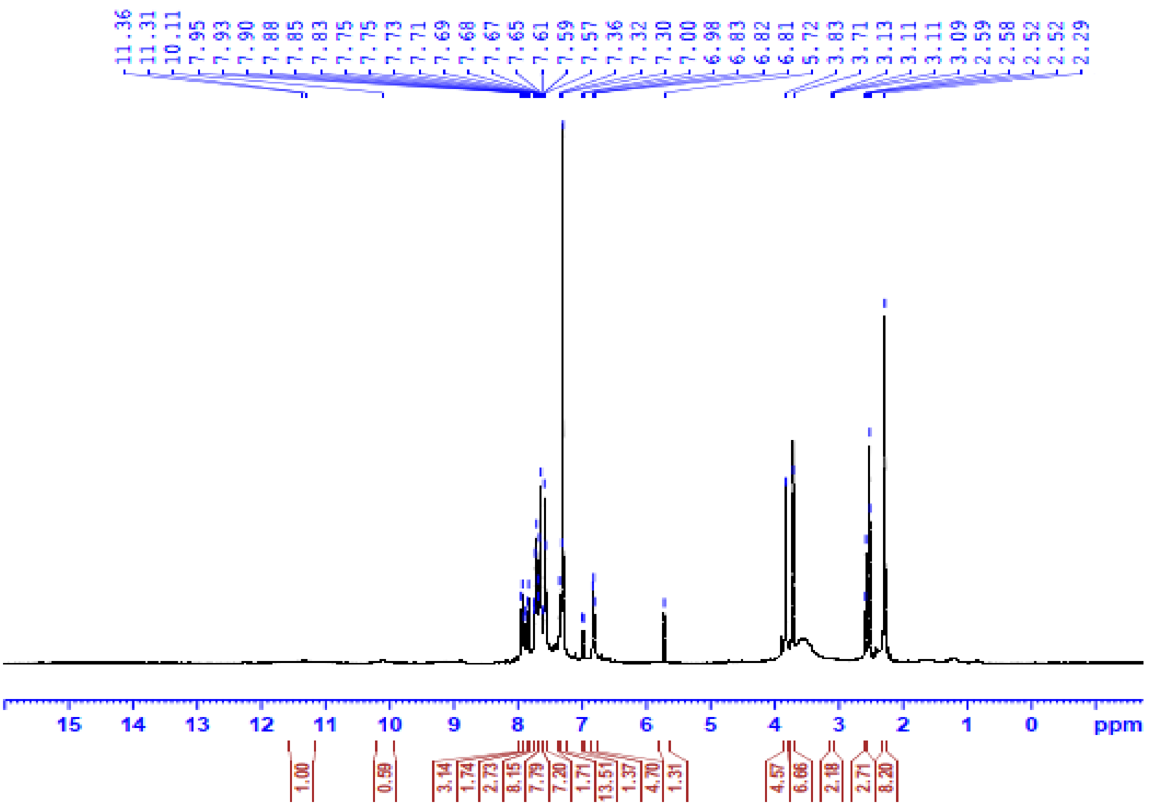

Figure S6a. ^{1}H-NMR spectrum of compound 5b

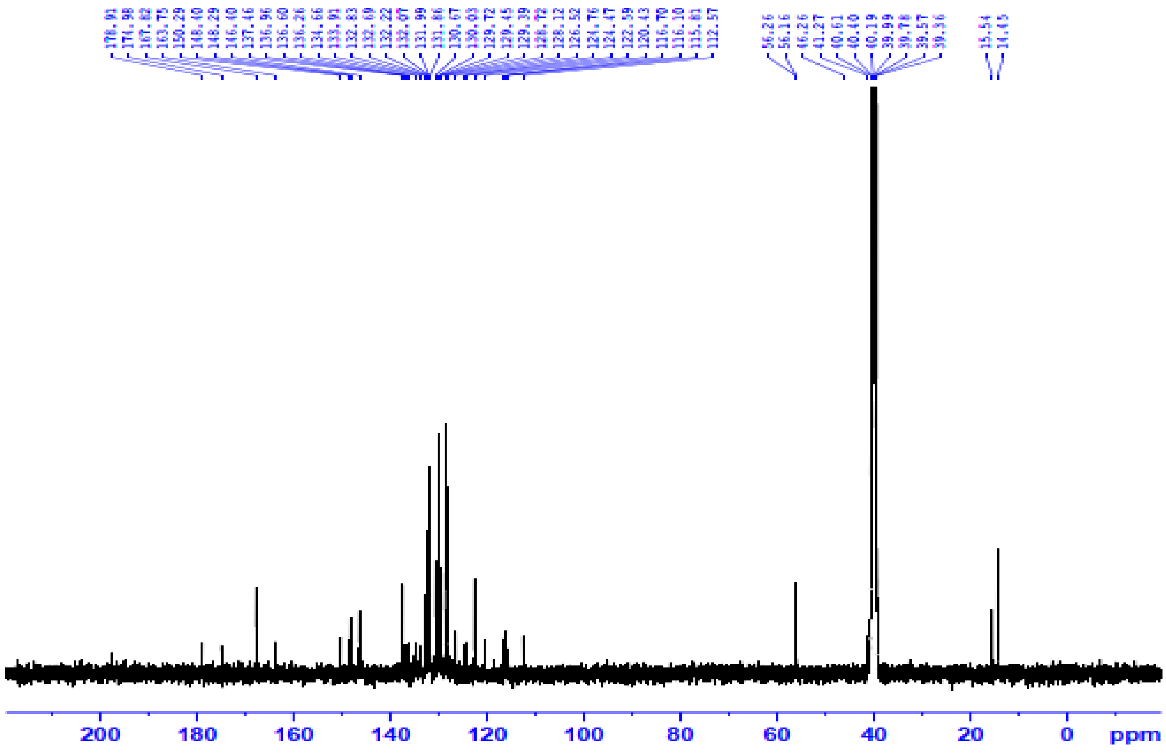

Figure S6b. ^{13}C-NMR spectrum of compound 5b.

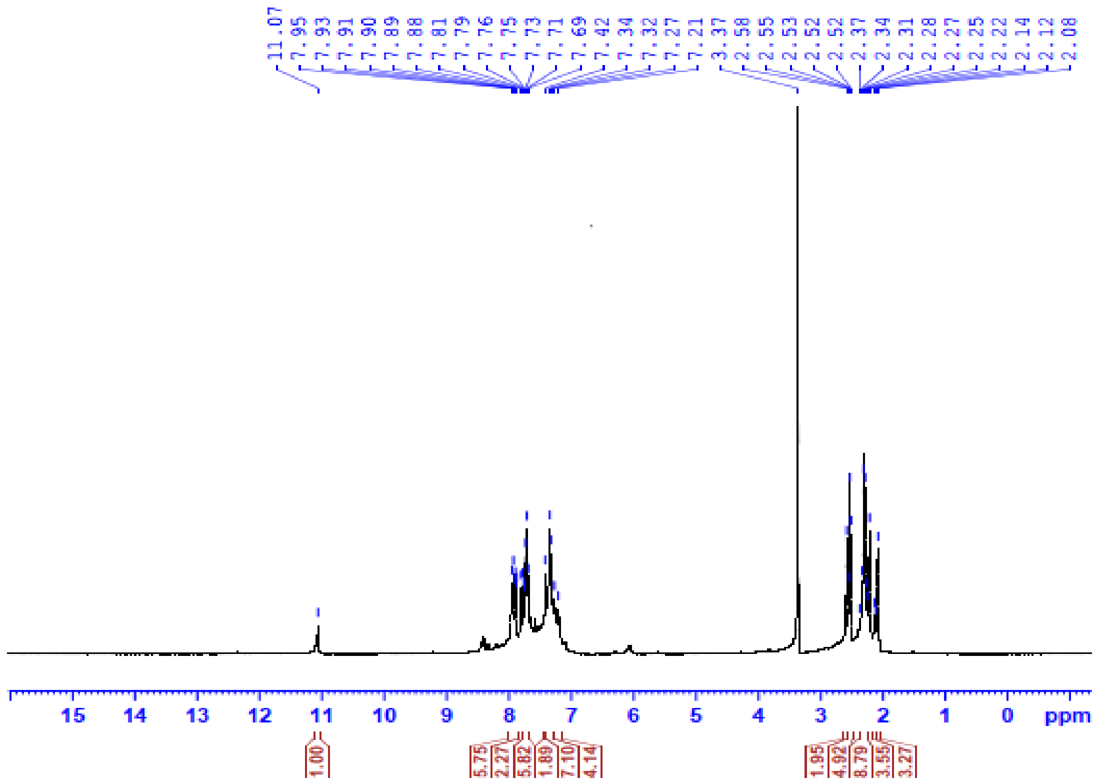

Figure S7a. ^{1}H-NMR spectrum of compound 6a

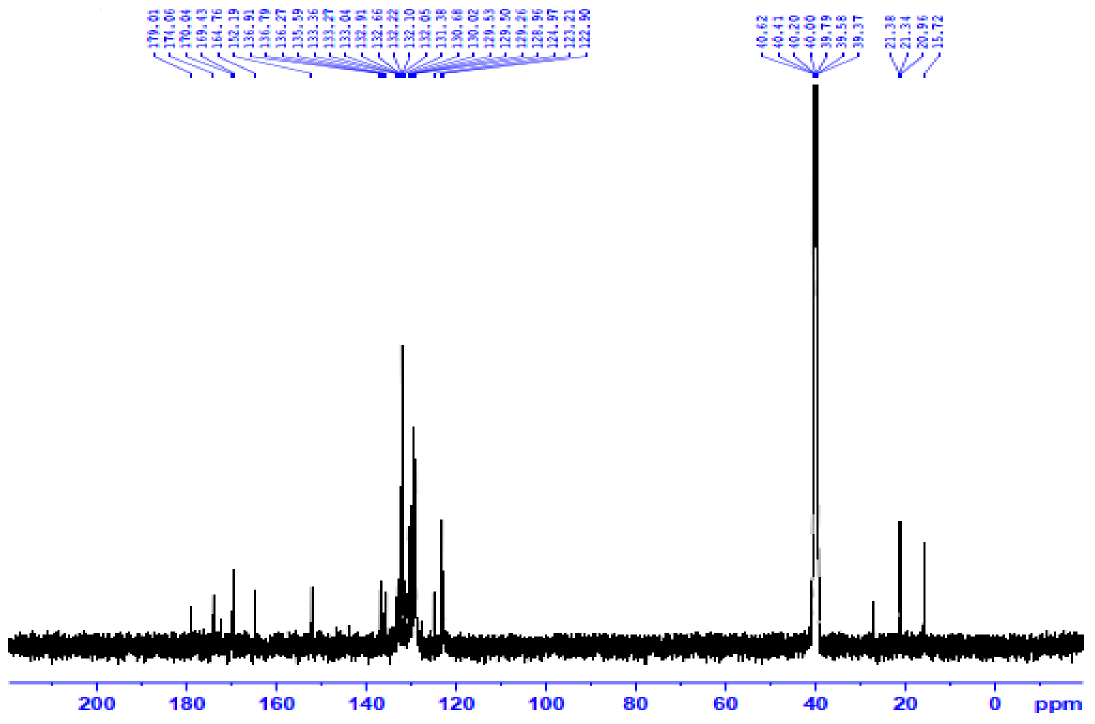

Figure S7b. ^{1}H-NMR spectrum of compound 6a

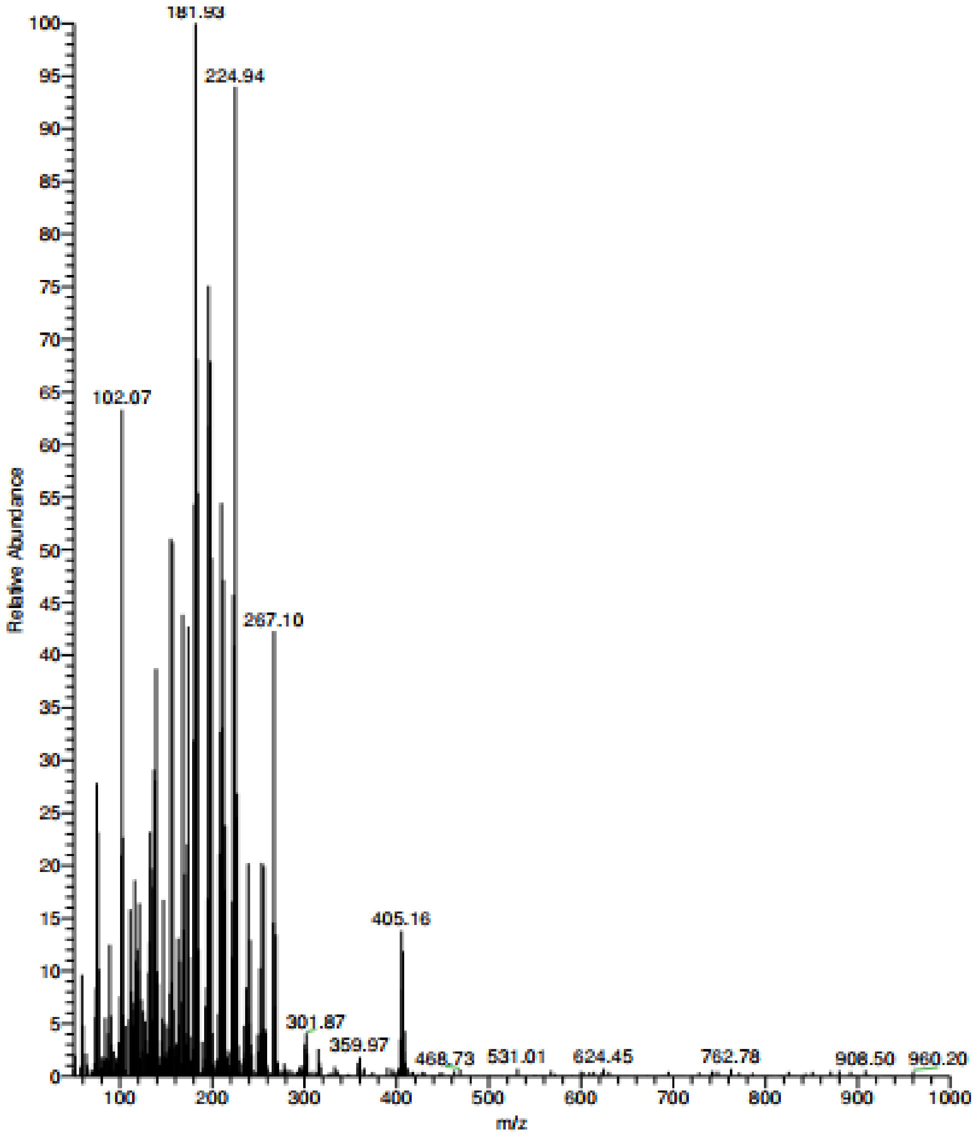

Figure S7c. Mass spectrum of compound **6a**

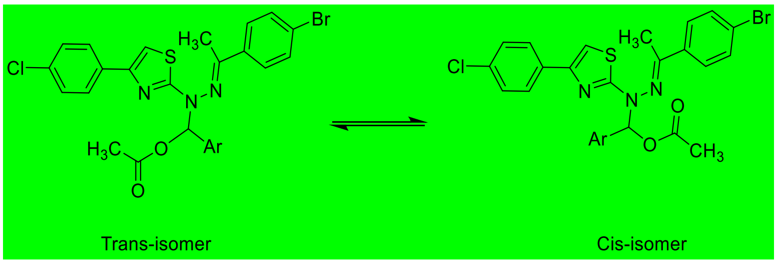

Figure S7d. Cis and Trans isomers of compounds **6a** and **6b**.

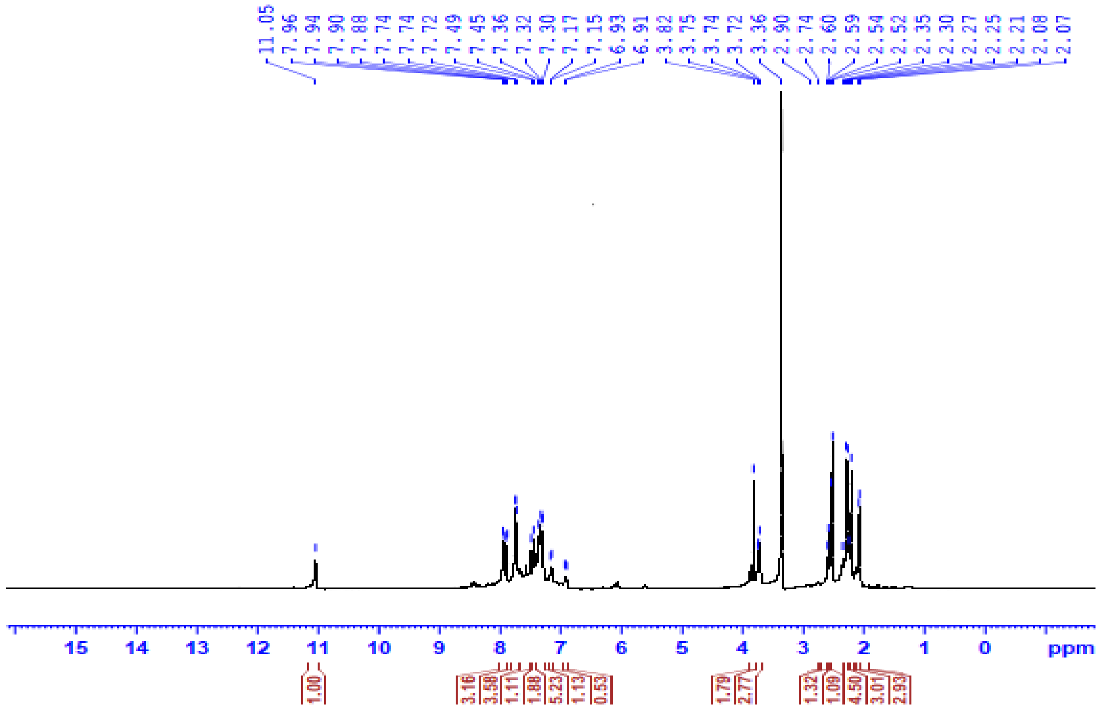

Figure S8a. [1]**H-NMR spectrum of compound 6b**

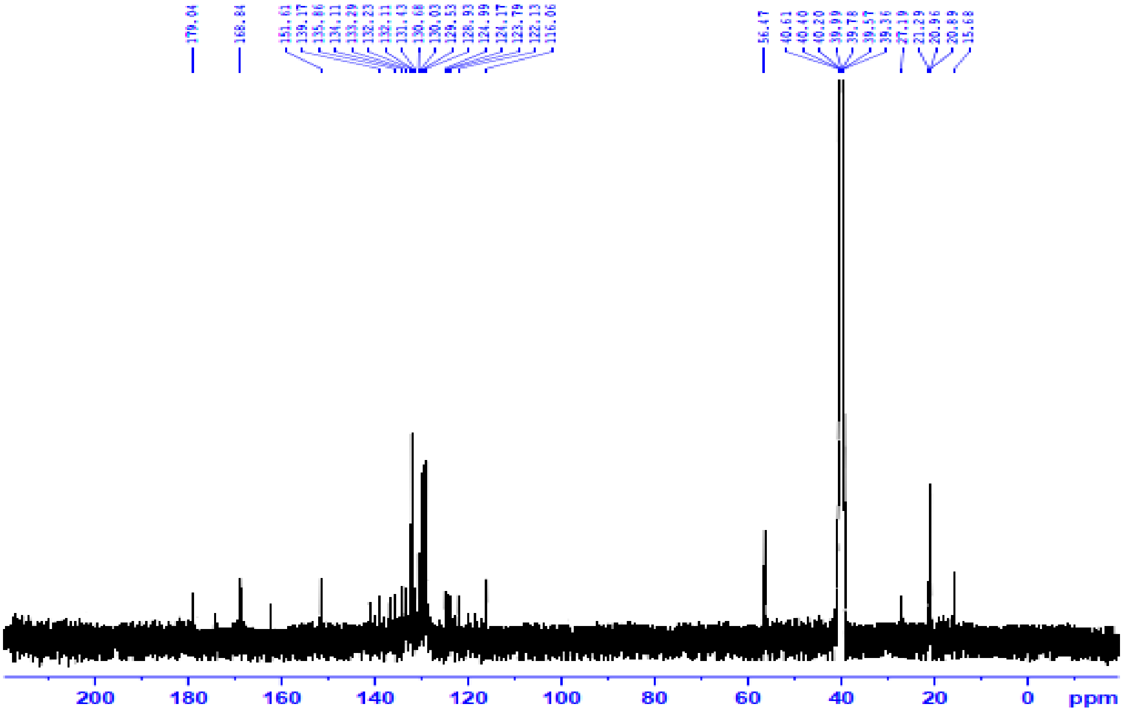

Figure S8b. [1]**H-NMR spectrum of compound 6b**

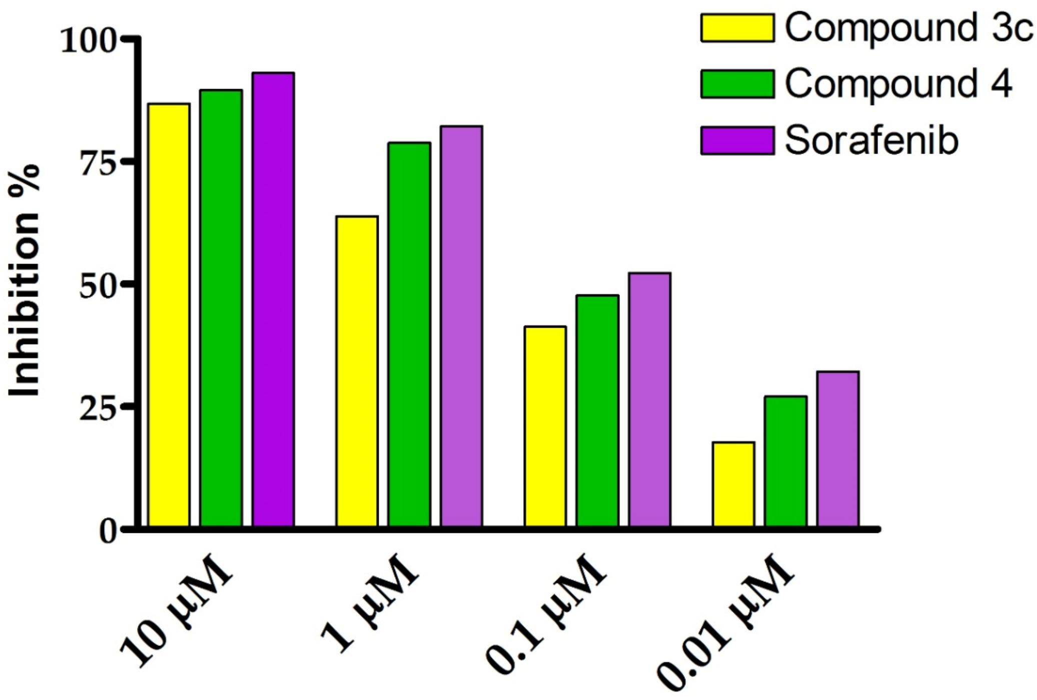

Figure S9. Inhibitory activity of compounds 3c, 4 and Sorafenib toward the VEGFR-2 kinase activity.
